# Genome-wide gene expression profiling of introgressed *indica* rice alleles associated with seedling cold tolerance improvement in a *japonica* rice background

**DOI:** 10.1186/z

**Published:** 2012-09-07

**Authors:** Fan Zhang, Liyu Huang, Wensheng Wang, Xiuqin Zhao, Linghua Zhu, Binying Fu, Zhikang Li

**Affiliations:** 1 Institute of Crop Sciences/National Key Facility for Crop Gene Resources and Genetic Improvement, Chinese Academy of Agricultural Sciences, Beijing 100081, China; 2 Shenzhen Institute of Breeding and Innovation, Chinese Academy of Agricultural Sciences, Shenzhen 518083, China; 3 International Rice Research Institute, DAPO Box 7777, Metro Manila, Philippines

**Keywords:** Rice, Cold tolerance, Transcriptome, Introgression line

## Abstract

**Background:**

Rice in tropical and sub-tropical areas is often subjected to cold stress at the seedling stage, resulting in poor growth and yield loss. Although *japonica* rice is generally more cold tolerant (CT) than *indica* rice, there are several favorable alleles for CT exist in *indica* that can be used to enhance CT in rice with a *japonica* background. Genome-wide gene expression profiling is an efficient way to decipher the molecular genetic mechanisms of CT enhancement and to provide valuable information for CT improvement in rice molecular breeding. In this study, the transcriptome of the CT introgression line (IL) K354 and its recurrent parent C418 under cold stress were comparatively analyzed to explore the possible CT enhancement mechanisms of K354.

**Results:**

A total of 3184 differentially expressed genes (DEGs), including 195 transcription factors, were identified in both lines under cold stress. About half of these DEGs were commonly regulated and involved in major cold responsive pathways associated with OsDREB1 and OsMyb4 regulons. K354-specific cold-induced genes were functionally related to stimulus response, cellular cell wall organization, and microtubule-based movement processes that may contribute to increase CT. A set of genes encoding membrane fluidity and defensive proteins were highly enriched only in K354, suggesting that they contribute to the inherent CT of K354. Candidate gene prediction based on introgressed regions in K354 revealed genotype-dependent CT enhancement mechanisms, associated with *Sir2*, *OsFAD7*, *OsWAK112d*, and programmed cell death (PCD) related genes, present in CT IL K354 but absent in its recurrent parent C418. In K354, a number of DEGs were co-localized onto introgressed segments associated with CT QTLs, providing a basis for gene cloning and elucidation of molecular mechanisms responsible for CT in rice.

**Conclusions:**

Genome-wide gene expression analysis revealed that genotype-specific cold induced genes and genes with higher basal expression in the CT genotype contribute jointly to CT improvement. The molecular genetic pathways of cold stress tolerance uncovered in this study, as well as the DEGs co-localized with CT-related QTLs, will serve as useful resources for further functional dissection of the molecular mechanisms of cold stress response in rice.

## Background

Rice (*Oryza sativa* L.) is an important cereal species, and a staple food for more than half of the world’s population. During early growth stages, low temperature stress retards rice seedling establishment and plant development, directly impacting yield. Temperate *japonica* cultivars generally have better seedling-stage cold tolerance (CT) and greater seedling vigor than *indica* cultivars [1]. By using backcross breeding, however, we have recently identified the putative genetic networks underlying some introgression lines (ILs) with significantly improved CT at the seedling stage under a *japonica* restorer C418 background (Zhang *et al.*, unpublished data), suggesting the diversity hidden in the *indica* germplasm. Further elucidation of the molecular basis of CT enhancement of *japonica* rice arising from introgression of favorable alleles from *indica* rice would provide rice breeders with much useful information.

Cold stress tolerance mechanisms in plants include cold signal perception, activation of transcription factors (TFs) by signal transduction, and expression of cold-responsive genes for mediating stress tolerance [2]. The plant cell membrane represents an ideal location for the primary temperature stress sensor, because one of the immediate effects of temperature stress in plants is a change in membrane fluidity [3]. At low temperatures, greater membrane lipid unsaturation appears to be crucial to optimum membrane function. When the *Arabidopsis* fatty acid deficient triple mutant *fad3-2 fad7-2 fad8*, which is devoid of trienoic fatty acids, was exposed to prolonged low temperatures, a decrease in fluorescence parameters, chlorophyll content, and thylakoid membrane content was observed compared with wild-type plants [4]. Similarly, *OsFAD8* has a functional role in maintaining levels of trienoic fatty acids and stress tolerance at low temperatures in rice [5]. In one study [6], linolenic acid levels increased and palmitic acid levels decreased in cold tolerant rice genotypes exposed to chilling; the opposite behavior was observed in cold sensitive genotypes. Transcriptional regulation is the core component of the complex genetic network associated with plant responses to cold. The C-repeat (CRT)/dehydration responsive element (DRE)-binding TF-mediated cold response pathway has been shown to play a predominant role in CT through the process of cold acclimation [7]. Although rice, a plant of tropical and subtropical origin, lacks mechanisms for cold acclimation, it nonetheless possesses components of this CBF cold-response pathway [8,9]. *OsDREB1A* and *OsDREB1B* are induced by cold stress, and constitutive overexpression of these genes in transgenic *Arabidopsis* and rice leads to induction of stress-responsive genes, increased high cold and salt tolerance, and growth retardation under normal conditions [8,9]. Other important signaling pathways have also been shown to be involved in CT. For example, *MYBS3* represses the DREB1-dependent cold signaling pathway at the transcriptional level. DREB1- and MYBS3-dependent pathways may complement each other and act sequentially to allow adaptation to immediate and persistent cold stress in rice [10]. The rice R2R3-type *OsMyb4* TF controls a hierarchical network comprising several regulatory sub-clusters associated with cellular defense and rescue, metabolism, and development. This network is independent of CBF/DREB, and its sub-regulons operate with possible co-regulators, including nuclear factor-Y [11]. In addition, one ROS (ROS-bZIP)-mediated regulon triggered by chilling stress is independent of Abscisic acid (ABA) and CBF/DREB, and its activation promotes rapid response of rice seedlings to chilling stress [12]. Finally, constitutive and non-cold responsive regulons, which have a differential effect on the cold responsive DRE regulon, also play a key role in CT [13].

DNA microarray analysis is a well developed high-throughput technology that has been used for many genomic application areas, especially whole-genome gene expression profiling. Although high-throughput RNA sequencing has recently become popular as an alternative to microarray analysis, the microarray platform, with its robust sample process and data analysis pipelines, is still the preferred choice for transcriptomic profiling involving a large number of samples in model plants with well-annotated genomes [14,15]. Many microarray-based studies have been carried out to identify abiotic stress responsive genes in specific rice varieties and transgenic rice [9,10,16,17], and comparative transcriptional profiling of two contrasting rice genotypes under salinity and drought stress have revealed novel genetic regulatory mechanisms involved in stress tolerance [18,19]. Unfortunately, it is difficult to use the stress-related genes uncovered in those studies to improve modern varieties in rice breeding, because most of them already exist in elite rice varieties.

In this study, a CT IL and its cold sensitive recurrent parent were assessed in terms of their seedling growth and physiological responses to cold stress treatment. An Affymetrix genome array was used to profile global gene expression changes under a cold stress time series. The results of this study should serve as an initial step in a comprehensive understanding of CT enhancement resulting from introgression of favorable alleles from *indica* rice into a *japonica* rice background.

## Results and Discussion

### Phenotypic differences between the introgression line K354 and its recurrent parent C418 under cold stress

The CT IL K354 and its recurrent parent C418, which possesses a cold sensitive phenotype, were used in this study. Under cold stress, a marked difference in survival rate was observed between K354 and C418 (Additional file 1), with K354 showing better CT and recovery ability than C418. Seedling leaves of C418 exhibited obvious wilting, while only a few necroses on leaf tips were observed in K354 seedlings (Additional file 2A). Compared with C418, cold-treated K354 seedlings experienced significantly less extensive cell membrane injury (relative electrolyte leakage) during the 24 h recovery period following the 48 h cold stress treatment (Additional file 2B). Interestingly, although under control conditions K354 exhibited significantly lower superoxide dismutase (SOD) activity and soluble protein concentrations than C418, after 48 h of cold stress these parameters increased dramatically and reached higher values, even with different changing trends, in K354 compared with C418 (Additional file 2C and 2E). In regard to catalase (CAT) activity, K354 showed a larger change than C418 after 48 h of cold stress (Additional file 2D). Based on phenotypic and physiological traits measured in this study, the CT of K354 was indeed improved over that of C418, due to the introgression of favorable alleles from *indica* donor Bg300 into the *japonica* background.

### Genome-wide gene expression profiling of K354 and C418 under a cold stress time-series

Comparison of genome-wide gene expression between two genotypes with contrasting CT phenotypes in a cold environment may help identify underlying molecular genetic pathways responsible for cold adaptation of rice plants. In this study, we use an Affymetrix whole rice genome array to profile gene expression changes of K354 and C418 under cold stress at five time points (2, 6, 12, 24, and 48 h). The array contained 49,824 rice genome genes (48,564 for *japonica* and 1260 for *indica*). Among these genes, 24,888–27,407 (43.4–47.8%) were found to be expressed in at least one sample in both genotypes under stress or control conditions (Additional file 3). A total of 3184 (5.6%) differentially expressed genes (DEGs) were identified, including comprise 2562 cold-responsive DEGs and 710 genotypic-specific DEGs (Table 1). There were 232/83, 286/243, 503/471, 1075/1092, and 1962/1444 DEGs detected in samples of C418/K354 at 2, 6, 12, 24, and 48 h, respectively, revealing that cold treatment induced a continuous increase in DEGs in both genotypes. There was an apparently delayed response to cold in K354 compared with C418, with 232 DEGs in C418 vs 83 in K354 at 2 h cold stress. Similar results were previously observed in rice under salinity stress; in that study, the sensitive genotype IR29 induced a relatively large number of genes compared to tolerant FL478 [18]. In addition, comparison of gene expression levels between K354 and C418 showed that there were 308 up-regulated and 209 down-regulated genes in K354 compared with C418 under control conditions. This suggests that these genes may be responsible for K354’s intrinsic tolerance to cold stress, which may be largely derived from introgression of donor alleles conferring constitutive tolerance to low temperature.

**Table 1:** Summary of cold-regulated genes and significant DEGs between two genotypes at the seedling stage

**Time course**	**Cold-responsive DEGs in C418**			**Cold-responsive DEGs in K354**			**K354 vs C418**		
	**Induced**	**Repressed**	**Sub-total**	**Induced**	**Repressed**	**Sub-Total**	**Up-**	**Down-**	**Sub-total**
Control	-	-	-	-	-	-	308	209	517
2 h	192	40	232	38	45	83	308	246	554
6 h	234	52	286	175	68	243	276	193	469
12 h	397	106	503	363	108	471	284	211	495
24 h	894	181	1075	932	160	1092	312	182	494
48 h	1436	526	1962	1151	293	1444	294	189	483

*Note:* DEGs were identified using the empirical criterion of more than 5-fold change and significant *t* tests (*p* < 0.05) based on three independent biological replicates. Induced or repressed genes were identified by comparing the transcription level of genes under stress condition with that under control conditions. Significantly up- or down-regulated DEGs between K354 and C418 were detected by comparing the transcription level of genes between two genotypes at the same time point.

**Figure 1:**
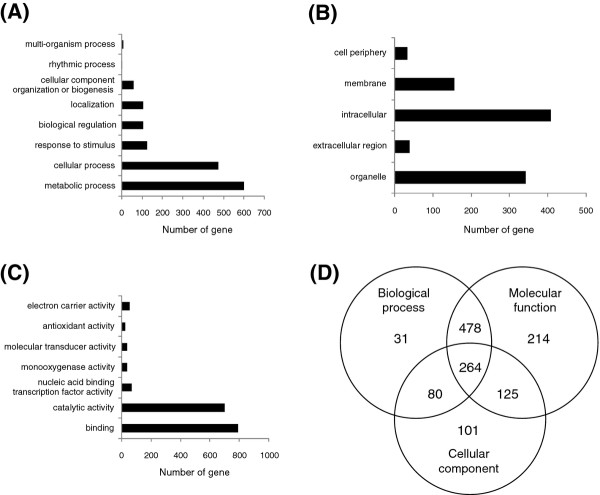
**Functional classification of all 2562 cold-responsive DEGs detected in C418 and K354. **(**A**) Biological processes; (**B**) cellular components; (**C**) molecular functions; (**D**) Venn diagram showing the overlap among the three categories. Bars show numbers of cold-induced genes in the two genotypes. Only significantly overrepresented GO slim categories are shown (*P* ≤ 0.05).

Gene Ontology (GO) enrichment of all detected cold-responsive DEGs with known and putative functions is shown in Figure 1. The predominant DEGs were functionally involved in metabolism (23.5%, *p* = 5.85E-33), such as macromolecule metabolism, oxidation-reduction, and primary metabolism; cellular processes (18.6%, *p* = 6.71E-11), including microtubule-based processes; stimulus response (4.9%, *p* = 1.04E-8); binding activity (31.0%, *p* = 5.26E-43), including ion, tetrapyrrole, and nucleotide binding; and catalytic activity (27.4%, *p* = 1.76E-43), such as hydrolase, transferase, and oxidoreductase activity. GO enrichment analysis of the 710 genotype-specific DEGs, however, indicated that the most prevalent DEGs were involved in programmed cell death (PCD) (3.2%, *p* = 4.68E-7) and electron carrier activity (3.0%, *p* = 0.0002), revealing genotype-specific transcriptome changes in response to cold environmental stimuli.

**Figure 2:**
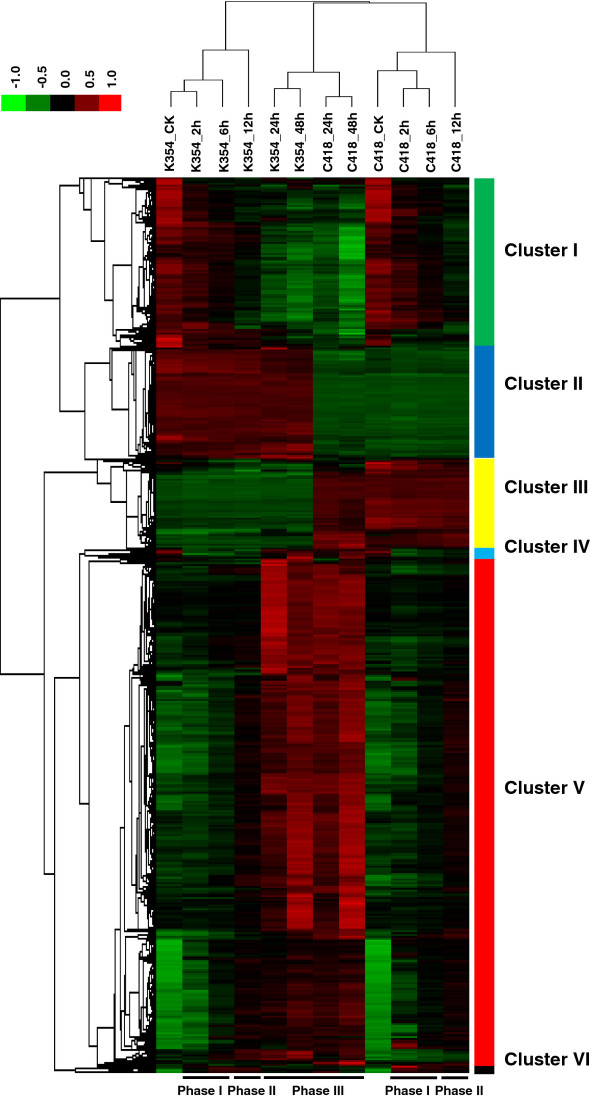
**Heat map of differentially expressed genes. **3184 DEGs (2562 cold-responsive DEGs and 710 genotypic-specific DEGs) identified in C418 and K354 under control and stress conditions were analyzed by hierarchical clustering. A heat map was constructed using the log-transformed and median-normalized expression values of the genes based on centered correlation distance and average linkage method. As a result, the 3184 genes were grouped into 6 clusters based on the genotypic-temporal pattern of gene expression. Relatively high expression values are shown in red.

On the basis of hierarchical average linkage cluster analysis (Figure 2), a transcript-level time-dependent cold response could be clearly delineated into early response (phase I, 2–6 h), middle response (phase II, 12 h), and late response (phase III, 24–48 h) components. The samples from K354 and C418 formed separate and distinct clusters showing their genotype-specific responses to cold stress treatment. All 3184 DEGs could be classified into six major groups based on their transcription patterns (Figure 2 and Additional file 4 and Additional file 5). Two major DEG groups shared by both genotypes were either induced (cluster V) and repressed (cluster I) by cold. Clusters II and III represented non-cold-responsive DEGs that were expressed at significantly different levels between C418 and K354 under normal growth conditions. Members of Cluster IV were initially down-regulated but later induced in both genotypes during cold stress. Interestingly, Cluster VI comprised a set of genes that were initially induced and later repressed in K354 during stress (Additional file 4 and Additional file 5). Genes in Clusters II, III, and VI may therefore be mainly responsible for the differential cold stress response between two genotypes, as discussed below.

Using the sample cluster results (Figure 2), 15 DEGs were randomly selected for qRT-PCR analysis to confirm the gene expression changes detected by microarray analysis. The high correlation (*R*^2^ = 0.75, *P* < 0.05) between microarray data and qRT-RCR expression values indicate that there was good agreement between both approaches (Additional file 6).

### Time-dependent K354 and C418 gene expression changes in response to cold stress

**Figure 3:**
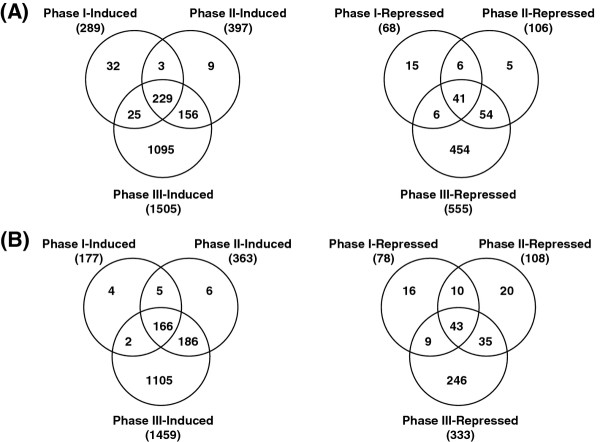
**Venn diagram of cold-regulated genes at different cold-responsive stages in C418 and K354. **(**A**) In C418 during phases I, II, and III; (**B**) In K354 during phases I, II, and III.

To assess the differential transcriptome alterations of K354 and C418 under cold stress, DEGs at different time points were compared by Venn diagram (Figure 3). Figure 3A represents the results of comparing induced or repressed genes in C418 under cold stress during phases I, II, and III. A total of 229 (14.8%) and 41 (7.1%) genes were continuously induced and repressed, respectively, in C418 during cold stress. Moreover, 32 (2.1%), 9 (0.6%), and 1095 (70.7%) genes were induced, and 15 (2.6%), 5 (0.9%), and 454 (78.1%) genes were repressed by cold in C418 specifically during phases I, II, and III, respectively. Similarly, a small proportion of genes were stably regulated by cold during phases I and II, but a larger number were exclusively regulated by cold during phase III in K354 (Figure 3B). These results indicate that in both genotypes most genes were regulated under later cold stress, implying that cold stress responsiveness increased with increasing treatment duration.

A total of 156 (78.8%), 303 (91.5%), and 1121 (61.4%) genes were commonly induced and 33 (40.0%), 56 (48.3%), and 248 (39.6%) were commonly repressed by cold in both genotypes during phases I, II, and III, respectively (Additional file 7). The large proportion of shared cold-regulated genes found in C418 and K354 suggests that the major genetic pathways associated with cold response are conserved in both genotypes, consistent with their mostly common genetic background, except for the introgressed genetic segments.

Furthermore, a set of 130 and 21 genes were identified as commonly induced and repressed genes, respectively, in both genotypes over the entire course of the cold treatment (Additional file 8). Among these, 28 TF genes from 9 families were induced, including 11 AP2-EREBP genes, 5 MYB genes, 3 WRKY genes, 3 ZIM genes, and 2 C2H2 genes. AP2-EREBP TFs, including *DREB1A* (Os09g35030), *DREB1B* (Os09g35010), *DREB1C* (Os06g03670), *DREB1F* (Os01g73770), *DREB1G* (Os02g45450), *ERF3* (Os01g0797600), and 5 other AP2 domain-containing proteins, were found to be significantly up-regulated in both genotypes under cold stress. This suggests that the major cold-responsive genetic pathway shared by C418 and K354 is possibly a DREB1-related pathway. In addition, we detected an important subset of commonly induced genes related to signal transduction in both genotypes, including genes encoding five MAPKKKs, four calmodulin-related calcium sensor proteins, and one CAMK. All these results indicate that conserved transcription regulation and signal transduction pathways are involved in cold stress response in both genotypes.

### Unique functional categories of genotype-specific DEGs in K354 and C418

To identify genotype-specific DEGs, we used the following criterion: any gene with more than 5-fold change found only in C418 or K354 at one or more cold-responsive stages was regarded as a C418 or K354 specifically-regulated gene set. After removing redundancies, 641 and 399 cold-responsive DEGs were specifically identified in C418 and K354, respectively (Table 2). GO analysis was performed using Zheng’s method [20] to functionally classify the genotype-specific DEGs regulated by cold and constitutive DEGs. There were 304 genes determined to be exclusively induced by cold stress in K354 (Additional file 9); their dominant GO categories were metabolic, cellular, stimulus response, and cellular component organization processes. The 95 K354-specific genes (Additional file 10) repressed by cold stress were largely involved in oxidation reduction processes and oxidative stress responses.

**Table 2:** List of cold-responsive and non-cold-responsive DEGs

**Cold-responsive DEGs**	**Induced**	**Repressed**	**Subtotal**
Common	991	249	1240
C418-specific	351	290	641
K354-specific	304	95	399
Non-cold-responsive constitutive DEGs			
Up-regulated in K354	-	-	184
Down-regulated in K354	-	-	112

In C418, 351 and 290 genes were identified as specifically induced and repressed under cold stress, respectively (Additional file 11 and Additional file 12). For the induced gene set, the most prevalent GO categories were oxidation-reduction (GO: 0055114), trehalose metabolic processes (GO: 0005991), and lipid transport (4 genes, GO: 0006869). Genes repressed exclusively by cold were involved in transmembrane transport (GO: 0055085), protein folding (GO: 0006457), oligopeptide transport (GO: 0006857), and L-methionine salvaging from methylthioadenosine (GO: 019509). The opposite transcription pattern observed for genes related to oxidation regulation can be partly explained by genetic differences in CT between the two genotypes. It has been suggested that oxidation regulation is involved in cold response [21], but whether high levels of antioxidant enzyme activity under cold stress are positively related to CT is presently unknown.

Among the constitutively highly expressed genes in K354 (Additional file 13), 184 genes were found to be non-responsive to cold stress, indicating an active role in CT in K354. All of these 184 genes were included in Cluster II (Figure 2). GO enrichment analysis indicated that these genes were functionally involved in endogenous stimulus (6.5%, *p* = 0.0006), protein modification (5.4%, *p* = 0.0013), signal transduction (5.4%, *p* = 0.0028), cell differentiation (2.2%, p = 0.0031), and amino acid and derivative metabolism (3.3%, *p* = 0.0035). Strikingly, ten genes with higher basal expression in K354 were related to PCD (GO: 0012501), and all of them were disease-resistance related genes with an NBS domain (Table 3). It has been reported that cell death pathways in plants can be activated not only by successful recognition of a pathogen during hypersensitive responses but also by a range of abiotic stresses [22]. In addition, PCD has been reported to be activated in tobacco cells in response to cold stress [23]. Furthermore, signal-induced plant PCD can be caused by reactive oxygen species generated by biotic and abiotic stresses, and can be perceived by the NBS domain [24]. In addition, in our study the rice omega-3 desaturase gene *OsFAD7* (Os03g18070) was 100 times more up-regulated in K354 than in C418 under control conditions. Rice has only one trienoic acid, linolenic acid, which is produced from linoleic acid by omega-3 fatty acid desaturase [5]. Increased linolenic acid levels are essential for the maintenance of membrane fluidity and chloroplast function at low temperatures [4]. Transgenic tobacco plants over-expressing *AtFAD7* have been reported to exhibit chilling tolerance [25]. We also observed that up-regulation of a cell wall-associated receptor kinase (OsWAK112d, Os10g10130) was 200 times greater in K354 than in C418 under control and cold stress conditions. Through an extracellular domain, wall-associated kinases physically link the plasma membrane to the cell wall matrix. They also have the potential to directly signal cellular events through their cytoplasmic kinase domain, which is one of the most likely candidates participating in cell wall-cytoplasm signaling in plant defense reactions [26]. All of these results suggest that the higher activity of defensive proteins and membrane fluidity under control conditions might be involved in CT in K354.

**Table 3:** Ten PCD and disease-resistance related non-cold-responsive genes with constitutively higher transcription levels in K354

**Gene ID**	**Annotation**	**Fold Change [K354] vs [C418]**					
		**Control**	**2 h**	**6 h**	**12 h**	**24 h**	**48 h**
LOC_Os11g37759	stripe rust resistance protein Yr10, putative, expressed	27.0	32.7	19.4	15.7	15.6	17.6
LOC_Os11g12340	disease resistance protein RPM1, putative, expressed	194.9	164.2	162.5	132.6	63.3	55.7
LOC_Os11g39320	LZ-NBS-LRR class, putative, expressed	19.2	19.3	11.0	9.9	9.2	8.0
LOC_Os06g15730	Disease resistance protein family protein.	15.9	14.0	11.6	11.2	10.9	16.6
LOC_Os11g39310	NB-ARC domain containing protein, expressed	18.8	19.9	13.4	12.6	10.5	15.2
LOC_Os11g39190	NB-ARC domain containing protein, putative, expressed	36.2	34.8	22.8	18.0	19.5	15.8
LOC_Os02g18070	NB-ARC domain containing protein, expressed	7.2	9.9	9.5	12.4	5.9	7.5
LOC_Os07g33730	NB-ARC domain containing protein, expressed	10.9	7.7	8.4	10.5	7.8	8.4
LOC_Os02g16270	xa1, putative, expressed	19.8	18.9	14.6	8.3	6.7	6.7
LOC_Os11g12000	NBS-LRR disease resistance protein, putative, expressed	26.3	19.5	20.0	20.6	24.3	39.0

### The DREB1 regulon plays an important role in cold stress response in both genotypes

Of the 2384 known or annotated TF genes in the rice gnome [27], 196 (8.2%) were observed to be differentially regulated by cold in this study (Additional file 14). These TF genes include 26 AP2/EREBP, 21 bHLH, 7 HSF, 19 MYB, 12 NAC, and 17 WRKY genes. A total of 16, 42, and 138 TF genes were identified as K354-specific, C418-specific, or commonly regulated by cold, respectively. Interestingly, five commonly regulated TFs (Os01g58420, Os04g49450, Os07g07974, Os09g33550, and Os11g08210 in Additional file 14) in both genotypes were previously reported to be regulated by cold-induced expression of *OsMyb4*[11], suggesting that a DREB/CBF-independent *OsMyb4* cold-regulated pathway exists in C418 and K354.

**Figure 4:**
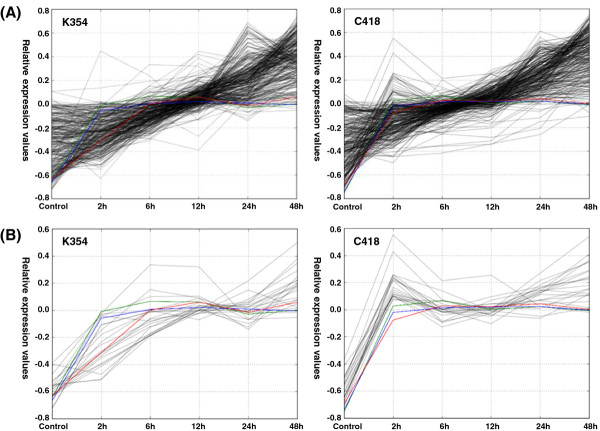
**Expression patterns of *OsDREB1 *and OsDREB1 regulon cold-responsive DEGs in K354 and C418. **Gene expression values during control conditions to 48 h after cold stress were log-transformed and median-normalized. (**A**) OsDREB1 regulon; (**B**) OsDREB1C-specific regulon. Black, green, blue and red lines indicate the OsDREB1 regulon, *OsDREB1A*, *OsDREB1B*, and *OsDREB1C*, respectively.

The OsDREB1 regulon has been shown to play an important role in conferring cold stress response in rice [28]. OsDREB1s belong to a sub-family of AP2/EREBP proteins unique to plants and sharing a highly conserved AP2 domain. To identify commonly cold-induced genes within the OsDREB1 regulon, we examined cis-regulatory elements in the 1-kb upstream regions of 991 commonly induced genes in both genotypes using the DRE core motif A/GCCGAC [29]. Of these genes, 298 (30.1%) contained at least one DRE core motif in the 1-kb upstream region (Additional file 15). As shown in Figure 4A, expression patterns of the OsDREB1 regulon (298 genes in Additional file 15) were roughly consistent with that of three OsDREB1 genes (*OsDREB1A*, *OsDREB1B*, and *OsDREB1C*). Of these 298 genes, 108 had been previously shown to be co-expressed with *OsDREB1* in other rice microarray experiments using a positive correlation coefficient of 0.9 as the cutoff on the TIGR website [30] (Additional file 15). *OsDREB1A* and *OsDREB1B* were induced by cold with a similar expression pattern but *OsDREB1C* was expressed differentially during phase I (2–6 h) between the two genotypes (Figure 4). Interestingly, analysis of gene expression profiles revealed the existence of an OsDREB1C-specific regulon, which included 22 genes exclusively regulated by *OsDREB1C*, (Figure 4B and Table 4) accounting for the main difference in the OsDREB1 regulon between two genotypes. These results indicate that the DREB1 regulon plays an important role in cold stress response in both genotypes, even though there are a few genes, such as the OsDREB1C regulon, whose regulation is unique to genotype-specific pathways.

**Table 4:** List of 22 genes of the OsDREB1C-specific regulon of commonly cold-induced DEGs between C418 and K354

**Gene ID**	**Annotation**	**Fold change [K354] vs [C418]**^**a**^			**Fold change [K354 stress] vs [K354 control]**		**Fold change [C418 stress] vs [C418 control]**		**Co-expressed with OsDREB1C (r > 0.9) in other microarray experiments available at TIGR website**^**b**^
		**Control**	**2 h**	**6 h**	**2 h**	**6 h**	**2 h**	**6 h**	
LOC_Os06g03670	OsDREB1C				6.08	35.80	33.09	60.14	
**OsDREB1 regulon**									
LOC_Os04g33820	OsFBX132 - F-box domain containing protein, expressed								GSE6901,GSE18361,E-MEXP-2267,E-MEXP-1766
LOC_Os01g17050	VQ domain containing protein, putative, expressed					5.46	8.04	5.32	GSE11025,GSE18361
LOC_Os01g48190	drought induced 19 protein, putative, expressed					6.53	8.53		
LOC_Os01g51670	expressed protein						13.96	7.19	GSE11025,GSE18361
LOC_Os01g52730	DUF584 domain containing protein, putative, expressed				6.27	8.36	9.59	7.32	GSE18361
LOC_Os01g56890	expressed protein						5.50		
LOC_Os01g58130	expressed protein						7.89		GSE18361
LOC_Os01g58140	expressed protein						9.10	6.98	GSE18361
LOC_Os01g64470	harpin-induced protein 1 domain containing protein, expressed					5.27	8.31	6.40	GSE11025,GSE18361,GSE17245
LOC_Os02g52210	zinc finger, C3HC4 type domain containing protein, expressed						5.95		
LOC_Os04g58890	expressed protein						8.68		GSE11025,GSE18361
LOC_Os05g03620	TKL_IRAK_CR4L.4 - The CR4L subfamily has homology with Crinkly4, expressed	0.19					6.76		GSE18361
LOC_Os05g31620	OsCML15 - Calmodulin-related calcium sensor protein, expressed				6.92	9.40	11.39	7.48	GSE18361,E-MEXP-1766
LOC_Os05g35500	MYB family transcription factor, putative, expressed	0.12					8.14		
LOC_Os05g39930	spotted leaf 11, putative, expressed						7.82	5.07	GSE11025,GSE18361
LOC_Os05g47960	expressed protein	0.18					15.73	5.76	GSE11025,GSE18361
LOC_Os06g13760	glycosyl transferase 8 domain containing protein, putative, expressed						5.47	5.44	
LOC_Os07g47790	AP2 domain containing protein, expressed	0.12					13.54	5.87	GSE18361
LOC_Os08g19670	expressed protein	0.20					7.90		GSE18361
LOC_Os08g31090	expressed protein						5.76		GSE11025,GSE18361
LOC_Os08g34580	trehalose-6-phosphate synthase, putative, expressed	0.09					11.36		E-MEXP-2267,E-MEXP-1766
LOC_Os09g37080	expressed protein	0.20				5.93	11.85	6.33	GSE11025,GSE18361,GSE6893,GSE17245

a. DEGs identified by using the empirical criterion of more than 5-fold change and cut-off p < 0.05 in SAM based on three independent biological replicates.

b. Experiment ID of other rice microarray experiments at TIGR website [30].

### Co-localization of cold-responsive DEGs and introgressed chromosome segments in K354

Based on the genotyping results, there were 17 introgressed chromosome segments in K354 that differed from C418 (Additional file 16). A number of quantitative trait loci (QTLs) related to CT have been mapped by different research groups [31-42], among them, 31 QTLs that were co-localized onto the 15 introgressed chromosome regions in K354. We correspondingly mapped 148 DEGs onto these introgressed regions (Additional file 17).

There were 32 DEGs co-mapped onto an introgressed region of chromosome 12 where two major CT QTLs were previously identified [31,42] (Additional file 17). Among these DEGs, two genes encoding the transcriptional regulator Sir2 protein (Os12g07950) and protein phosphatase 2C were highly enriched in K354. According to GO analysis, Sir2 is related to epigenetic function (GO: 0040029), and the protein phosphatase 2C is involved in stress signaling [43]. These two genes are candidates for the CT QTL. In addition, most of the DEGs co-localized on the introgressed segment of chromosome 2 were found to be functionally associated with stress response. These genes include genes encoding cytochrome P450s, terpene synthase, NB-ARC domain containing protein, histone deacetylase, and TF *OsWRKY42*. Importantly, there was only one candidate gene, *OsFAD7*, found in the introgressed region near RM251 on chromosome 3 (Additional file 17). *OsFAD7* has been reported to be highly induced by extreme temperature, and is involved in membrane stability [44], indicating it plays a crucial role in maintaining cell membrane fluidity in K354 under cold stress. DEGs on the introgressed regions of RM262 on chromosome 2, RM432 on chromosome 7, and RM38 on chromosome 8 were largely related to signal transduction cascades. These genes included genes encoding terpene synthase (Os02g36140, Os08g07100), protein phosphatase 2C (Os07g32380), and receptor-like kinase OsSIK1 (Os08g02996), which are known to be strongly associated with abiotic stress response [43,45,46]. Strikingly, a gene cluster containing four PCD-related genes were co-localized on the introgressed region of chromosome 11 (Additional file 17), indicating their distinct role in CT of K354. These DEGs may serve as functional candidate genes for the identification of a QTL for CT. By combining further functional identification and QTL fine mapping, the co-localized DEGs detected in this microarray analysis may provide the basis for gene cloning and elucidation of the molecular mechanisms responsible for CT in rice.

One objective of this study was an attribution of the differences between C418 and K354 transcriptomes to differences in CT between the two genotypes. These differences, whether at the transcription or phenotypic level, can only be caused by introgressed fragments from Bg300, an *indica* variety. Although *japonica* varieties are generally known to be more tolerant to cold stress than *indica* varieties, we have shown in this study that some CT alleles are “hidden” in the *indica* background. This means that the CT effect of the alleles may not be favorable in *indica*, but when introgressed into a different background (i.e., *japonica*), the allele may manifest a different pattern. Because the genomes of C418 and K354 are almost identical, any transcriptomic differences between the two genotypes must be due to the presence of fragments introgressed from Bg300, whether caused by direct allelic replacement or genome-wide transcriptome reconfiguration as a result of the replacement. Comparison of C418 and K354 transcriptomes can therefore reduce background noise from Bg300. Although we did not do so in this study, the question of which process, i.e., direct allelic replacement or genome-wide transcriptome reconfiguration, is responsible for transcriptome changes between C418 and K354 could be resolved by adding Bg300 to the transcriptome comparison.

Many K354-specific expressed genes that were not mapped onto or near introgressed regions may also play a vital role in CT, possibly through regulation by co-localized DEGs. By this we mean that a large number of genes involved in a signaling pathway related to a certain trait may be activated by expression of upstream genes. Due to the low resolution of the limited SSR markers, we can only roughly estimate the length of the introduced fragments. It is worth noting, however, that whole genomic re-sequencing for K354 and C418 is underway. We believe the re-sequencing results will provide more detailed and precise information regarding the introgressed chromosomal segments. Fine-mapping of these segments is necessary to more precisely identify the functional candidate genes.

## Conclusions

Using expression profiling with an Affymetrix rice genome array, we found that at the seedling stage the CT IL K354 and its recurrent parent C418 exhibited a diverse whole genome-wide transcriptional response under both control and cold stress conditions. We detected 3184 genes that were differentially regulated under cold stress, accounting for approximately 6% of total genes on the rice microarray chip. A large proportion of genes, including DREB1 and OsMyb4 regulons, were determined to be commonly regulated by cold in both genotypes. Expression of cold-responsive genes specific to the cold-tolerant genotype K354 may be affected by introgressed chromosome segments, and these genes may contribute to the increased CT of K354. Among genes co-localizing with introgressed chromosomal segments from the *indica* donor parent were genes involved in PCD, which were constitutive but had higher absolute expression levels in the IL than in the *japonica* recurrent parent, and some differentially induced genes likely to be involved in the regulation of membrane biogenesis. Our results represent a preliminary elucidation of genotype-dependent CT enhancement mechanisms, and may contribute to the improvement of CT in *japonica* rice using *indica* germplasm. Functional validation of CT related genes identified in this study, however, is still required to provide more useful information for CT improvement in *japonica* rice molecular breeding.

## Methods

### Plant materials and cold treatment

A *japonica* restorer line, C418, which is the male parent of many elite *japonica* commercial hybrid cultivars widely grown in northern China, and the CT IL K354, a BC_2_F_6_ IL derived from a cross between recurrent parent C418 and Bg300 (an *indica* variety from Sri Lanka), were used in this study. After an extensive study using 100 simple sequence repeat (SSR) markers that were polymorphic between C418 and Bg300, selected from 600 SSR markers across the rice genome [47], K354 was found to differ from C418 at 17 genomic segments derived from Bg300 (Additional file 16).

Sterilized seeds of C418 and K354 were allowed to germinate in distilled water for 2 d. Well-germinated seeds were sown in soil-filled plates with holes at the bottom. C418 and K354 were planted at the same spacing in the same plates. The plates were placed in a 65 cm × 44 cm × 14 cm plastic case with nutrient solution, following the IRRI standard protocol [48], and transferred to a growth chamber (Beijing ZNYT, China). Both cold stress and control treatments were carried out with 24 replicates for each line. Approximately 15 vigorous seedlings from each replicate were allowed to grow until the three-leaf stage in the growth chamber. Growing conditions were a constant 25°C day/night temperature, 12 h photoperiod, and 75–80% relative humidity.

At the three-leaf stage, seedlings were transferred to a 4°C chamber. The time point at which seedlings were placed in the 4°C chamber was defined as 0 h. Whole shoot samples were collected at 2, 6, 12, 24, and 48 h and frozen immediately in liquid nitrogen. Control seedlings were not exposed to cold stress, but were instead maintained at a constant temperature of 25°C; whole shoot samples were taken from the controls at 2 h and 24 h. Collections at the two time points were pooled together as a control. Three biological replicates (each from a separate plastic pot) were prepared for microarray analysis. After collection, samples were kept in a −80°C freezer for later total RNA extraction. To detect phenotypic changes in the stressed seedlings, seedlings were moved to control conditions after 48 h of stress. After 24 h recovery, a photograph of each seedling was taken to serve as a phenotypic record.

### Physiological traits of the two genotypes under cold stress

Membrane stability was measured using a previously published procedure [49], with minor modifications for rice leaf tissue. Three replicates of 0.5 g fresh leaves were sampled from control and cold-treated seedlings. After being cut into 1-cm pieces, the 0.5 g leaf samples were immersed in 20 mL distilled water in a test tube for 1 h with the aid of a vacuum pump. After standing for 2 h at 25°C, water conductivity was measured. Leaf discs were then killed in the same solution by autoclaving, and total conductivity was measured at room temperature. Percent injury arising from each treatment was calculated from conductivity data using the equation:% injury = [(% L(t) -% L(c))/(100-% L(c))] × 100), where% L(t) and% L(c) are percent conductivity for treated and control samples, respectively. Antioxidant enzyme activity, including that of SOD and CAT, were determined following previously reported methods [50]. Soluble protein concentrations were measured according to the protocol of Bradford [51].

### RNA extraction and processing for microarray analysis

RNA was prepared following the recommendations in the Affymetrix GeneChip Expression Analysis technical manual. Briefly, total RNA was isolated from samples frozen in liquid nitrogen using TRIZOL reagent, and then processed by CapitalBio Corporation (Beijing, China) according to the following steps. Two micrograms of purified total RNA were used to synthesize double-stranded cDNA. Biotin-tagged cRNA was generated from an *in vitro* transcription reaction using a MessageAmp II aRNA amplification kit and then fragmented into 35–200 bp long strands following the Affymetrix protocol. The cRNA was then hybridized to an Affymetrix rice genome array containing 48,564 *japonica* and 1260 *indica* sequences at 45°C with rotation for 16 h in an Affymetrix GeneChip 640 hybridization oven. The GeneChip arrays were washed and stained on an Affymetrix 450 Fluidics Station, and then scanned using an Affymetrix 3000 GeneChip Scanner.

### Array data statistical analysis

GeneChip Operating Software (GCOS1.4) was used to analyze the hybridization data. Following visual inspection of scanned images, satisfactory images were analyzed to generate CEL raw data files using GCOS1.4 default settings. dChip software was used to perform array normalization, following the dChip user’s manual, according to an invariant set approach. For the comparison analysis, SAM (Significant Analysis of Microarray) software was applied using the two-class unpaired method to identify DEGs between cold-stressed samples and control samples for a given genotype, and between two genotypes at each time point. As there is not a fixed standard threshold for significant differential gene expression, we identified DEGs using the empirical criterion of more than 5-fold change and a cut-off of *p* < 0.05 in SAM based on three independent biological replicates. An average linkage hierarchical cluster analysis was performed on the DEGs using GeneCluster version 3.0 software [52].

The putative function of each DEG corresponding to a probe on the Genechip was predicted based on its Affymetrix annotation combined with the TIGR definition [30]. GO enrichment analysis was performed using the web-based software toolkit GOEAST [20]. To analyze DEG regulatory elements, promoter regions (−10 to −1000 bp upstream of the start codon) of selected DEGs were scanned for the DRE motif A/GCCGAC using a Perl program from CapitalBio Corporation. Gene sequences, including 1 kb upstream sequences, of all selected DEGs were downloaded from the TIGR website [30].

### Quantitative RT-PCR validation of DEGs

A total of 15 genes were selected for qRT-PCR validation of the expression level changes identified in the microarray analysis. The selected genes included OsDREB1 regulon genes and genes that were significantly differentially expressed between K354 and C418 and near or within regions of donor introgressed fragments in K354. Sequences corresponding to the selected genes were downloaded from the TIGR rice database [30], and Primer 5.0 software was then used to design qRT-PCR primers based on exonic gene regions. An *Actin* gene was used as the endogenous control. First-strand cDNA was synthesized by reverse transcription using 2 μg of total RNA in a 100 μL reaction mixture using a Reverse Transcription System (Promega, A3500). Diluted synthesized cDNA (1 μL) was used for qRT-PCR analysis along with 200 nM of each primer mixed with SYBR Green PCR Master Mix (Takara Code: DRR041A). qRT-PCR was performed in triplicate for each data point using an ABI7300 sequence detection system and the same RNA samples used for the microarray analysis. Relative expression values were calculated after normalizing against the maximum expression value. The data were further normalized to facilitate profile matching to data obtained from the microarray experiments.

### Data availability

The entire set of original microarray data has been deposited in NCBI’s Gene Expression Omnibus [53] under GEO Series number GSE37940.

## Abbreviations

CT: Cold tolerance; IL: Introgression line; DEGs: Differentially expressed genes; TF: Transcription factors; ABA: Abscisic acid; SOD: Superoxide dismutase; CAT: Catalase; PCD: Programmed cell death; GO: Gene Ontology; QTLs: Quantitative trait loci; SSR: Simple sequence repeat; SAM: Significant analysis of microarray.

## Competing interests

The authors declare that they have no competing interests.

## Authors’ contributions

FZ, BF and ZL designed the experiments and drafted the manuscript. FZ, LH, WW, XZ and LZ conducted the phenotype and microarray experiments. FZ performed the microarray data analysis, and LH carried out the qRT-PCR confirmation of candidate genes. BF revised the manuscript. All authors read and approved the final manuscript.

## Supplementary Material

Additional file 1 **Phenotypic performance between C418 and K354 in different experiments. **Excel file containing the phenotypic performance of C418 and K354 under both control and abiotic stress conditions in different experiments. Click here for file

Additional file 2 **Phenotype and physiological changes of K354 and C418 under control and cold stress conditions.** A PowerPoint file containing photographs of growth status and physiological conditions of K354 and C418 under control conditions (25°C) and cold stress treatment (4°C) for 48 h. Click here for file

Additional file 3 **Total number of expressed transcripts. **Word file containing the total number of transcripts expressed in the two genotypes at different times under control and cold stress conditions. Click here for file

Additional file 4 **Expression patterns of differentially expressed transcripts in K354 and C418. **A PowerPoint file containing expression patterns of 3184 differentially expressed probes based on log-transformed and normalized expression values from control conditions to 48 h under cold stress in (A) K354 and (B) C418. Green, blue, yellow, cyan, red, and black lines indicate clusters I–VI with the same color in Figure 2, respectively. Click here for file

Additional file 5 **Detailed list of DEGs in the six clusters of Figure**2 Excel file containing a list of the transcripts used in the cluster analysis in Figure 2. Click here for file

Additional file 6 **Validation of gene expression by qRT-PCR. **A PowerPoint file containing correlation analysis results between microarray and qRT-RCR experiments based on 15 selected genes representing the OsDREB1 regulon and candidate genes of introgressed segments. Gene expression values were transformed to a log_2_ scale. The microarray data log_2_-value (*X*-axis) were plotted against the qRT-PCR log_2_-value (*Y*-axis). Click here for file

Additional file 7 **Comparative diagram of the total number of differentially regulated genes between C418 and K354 under cold stress.** A PowerPoint file containing (A) Cold-induced genes; (B) Cold-repressed genes. Blue, red, and green bars indicate K354-specific (blue bar), common (red bar), and C418-specific (green bar) regulated genes by cold stress at phase I (2–6 h), phase II (12 h), and phase III (24–48 h) of cold-response, respectively. Click here for file

Additional file 8 **Common genes regulated by cold in both genotypes at all cold-responsive stages. **Word file containing 130 and 21 genes commonly induced and repressed by cold in both genotypes at all cold-responsive stages. Click here for file

Additional file 9 **K354-specific cold-induced genes. **Excel file containing a list of K354-specific cold-induced genes at all time points. Click here for file

Additional file 10 **K354-specific cold-repressed genes. **Excel file containing a list of K354-specific cold-repressed genes at all time points. Click here for file

Additional file 11 **C418-specific cold-induced genes. **Excel file containing a list of C418-specific cold-induced genes at all time points. Click here for file

Additional file 12 **C418-specific cold-repressed genes. **Excel file containing a list of C418-specific cold-repressed genes at all time points. Click here for file

Additional file 13 **Constitutively highly-expressed genes in K354. **Excel file containing a list of non-cold-responsive genes with constitutively higher transcriptional expression in K354 than in C418. Click here for file

Additional file 14 **TF genes detected to be differentially regulated by cold. **Excel file containing a list of TF genes differentially regulated by cold in K354 and C418. Click here for file

Additional file 15 **OsDREB1s and commonly cold-induced genes including a DRE core motif. **Excel file containing a list of OsDREB1s and 298 genes commonly cold-induced in both C418 and K354 with at least one DRE core motif at 1 kb up-stream. Click here for file

Additional file 16 **Genomic distribution of 17 introgression fragments in K354.** A PowerPoint file containing information on introgressed fragments, polymorphic SSR markers used, and previously reported QTLs related to CT at the seedling stage near the introgressed regions. Click here for file

Additional file 17 **DEGs in the introgressed regions of K354. **Excel file containing a list of differentially regulated genes detected by comparison of gene expression profiles of two genotypes that were within or neighboring the regions of donor introgression. Click here for file
