# Supplementary material for: Genome-wide gene expression profiling of introgressed indica rice alleles associated with seedling cold tolerance improvement in a japonica rice background
Source: BMC Genomics. 2012 Sep 7;13:461. doi: 10.1186/z (PMC3526417; doi:10.1186/z)
Supplement: Additional file 2 — Phenotype and physiological changes of K354 and C418 under control and cold stress conditions. A PowerPoint file containing photographs of growth status and physiological conditions of K354 and C418 under control conditions (25°C) and cold stress treatment (4°C) for 48 h. [file 1471-2164-13-461-S2.ppt]

## Slide 1
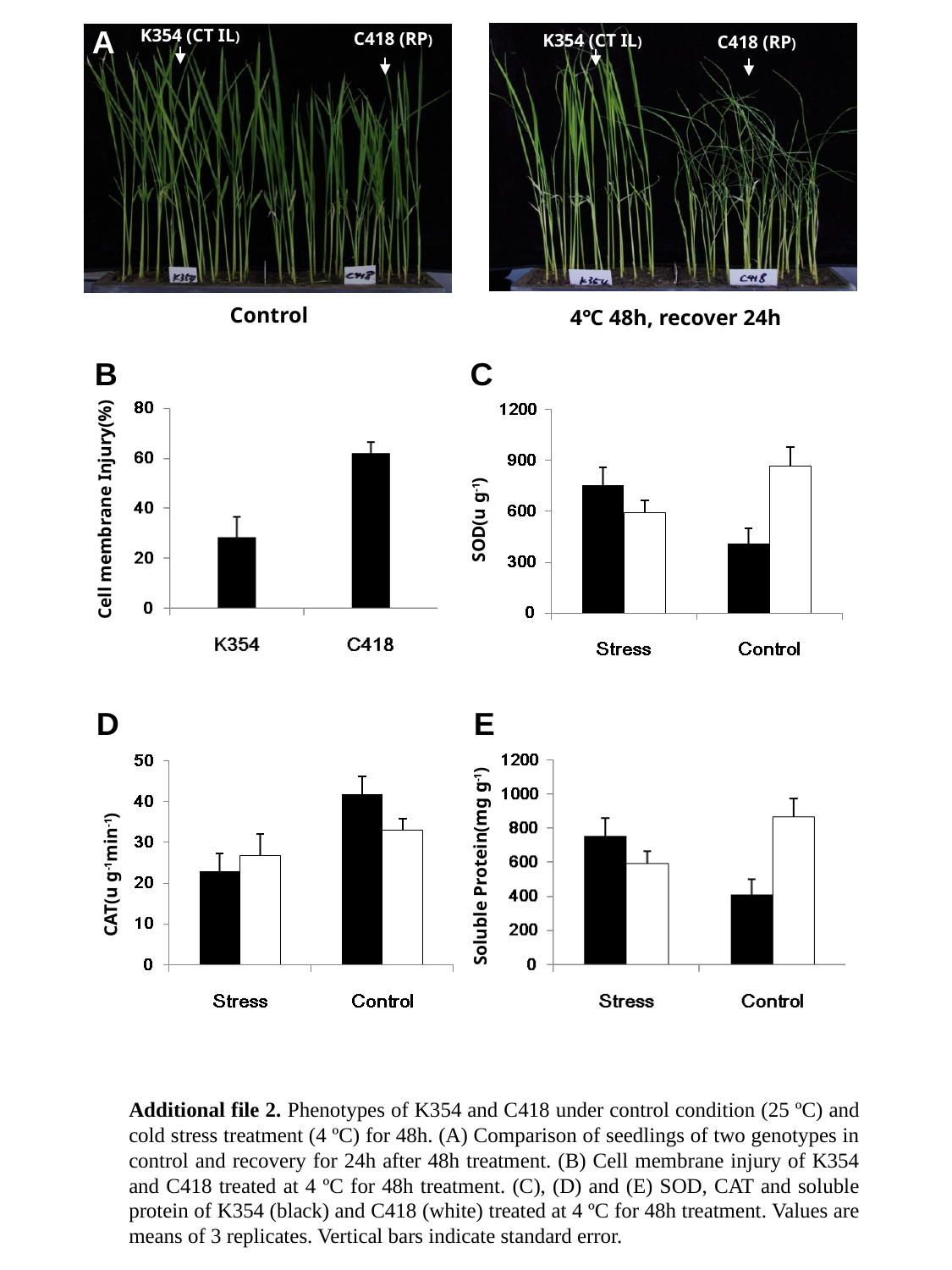

A
K354 (CT IL)
C418 (RP)
K354 (CT IL)
C418 (RP)
4℃ 48h, recover 24h
Control
B
C
Cell membrane Injury(%)
SOD(u g-1)
D
E
CAT(u g-1min-1)
Soluble Protein(mg g-1)
Additional file 2. Phenotypes of K354 and C418 under control condition (25 ºC) and cold stress treatment (4 ºC) for 48h. (A) Comparison of seedlings of two genotypes in control and recovery for 24h after 48h treatment. (B) Cell membrane injury of K354 and C418 treated at 4 ºC for 48h treatment. (C), (D) and (E) SOD, CAT and soluble protein of K354 (black) and C418 (white) treated at 4 ºC for 48h treatment. Values are means of 3 replicates. Vertical bars indicate standard error.
