# Supplementary material for: Genome-wide gene expression profiling of introgressed indica rice alleles associated with seedling cold tolerance improvement in a japonica rice background
Source: BMC Genomics. 2012 Sep 7;13:461. doi: 10.1186/z (PMC3526417; doi:10.1186/z)
Supplement: Additional file 3 — Total number of expressed transcripts. Word file containing the total number of transcripts expressed in the two genotypes at different times under control and cold stress conditions. [file 1471-2164-13-461-S3.doc]

**Additional file 3**. Total number of probes expressed of two genotypes at different time course under control and cold stress conditions.

| Time Ponits | C418 | Percentage% | K354 | Percentage% |
| --- | --- | --- | --- | --- |
| Control | 26029 | 45.4 | 25284 | 44.1 |
| 2h | 26189 | 45.6 | 26419 | 46.0 |
| 6h | 25556 | 44.5 | 25335 | 44.2 |
| 12h | 25456 | 44.4 | 24888 | 43.4 |
| 24h | 26604 | 46.4 | 27407 | 47.8 |
| 48h | 26673 | 46.5 | 25618 | 44.6 |
