# Supplementary material for: Genome-wide gene expression profiling of introgressed indica rice alleles associated with seedling cold tolerance improvement in a japonica rice background
Source: BMC Genomics. 2012 Sep 7;13:461. doi: 10.1186/z (PMC3526417; doi:10.1186/z)
Supplement: Additional file 4 — Expression patterns of differentially expressed transcripts in K354 and C418. A PowerPoint file containing expression patterns of 3184 differentially expressed probes based on log-transformed and normalized expression values from control conditions to 48 h under cold stress in (A) K354 and (B) C418. Green, blue, yellow, cyan, red, and black lines indicate clusters I–VI with the same color in Figure 2, respectively. [file 1471-2164-13-461-S4.ppt]

## Slide 1
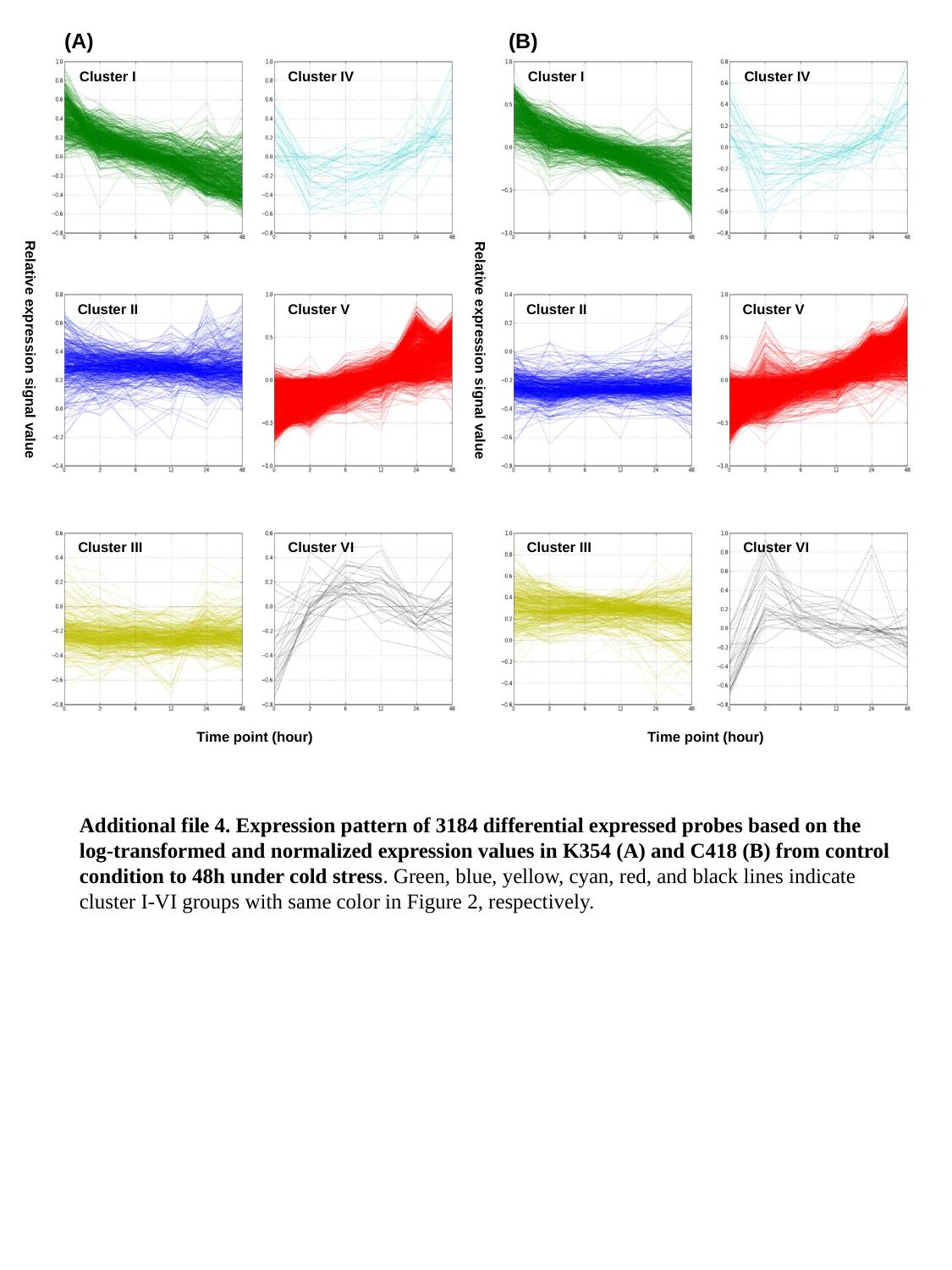

(A)
(B)
Cluster I
Cluster IV
Cluster I
Cluster IV
Cluster II
Cluster V
Cluster II
Cluster V
Relative expression signal value
Relative expression signal value
Cluster III
Cluster VI
Cluster III
Cluster VI
Time point (hour)
Time point (hour)
Additional file 4. Expression pattern of 3184 differential expressed probes based on the log-transformed and normalized expression values in K354 (A) and C418 (B) from control condition to 48h under cold stress. Green, blue, yellow, cyan, red, and black lines indicate cluster I-VI groups with same color in Figure 2, respectively.
