# Supplementary material for: Genome-wide gene expression profiling of introgressed indica rice alleles associated with seedling cold tolerance improvement in a japonica rice background
Source: BMC Genomics. 2012 Sep 7;13:461. doi: 10.1186/z (PMC3526417; doi:10.1186/z)
Supplement: Additional file 6 — Validation of gene expression by qRT-PCR. A PowerPoint file containing correlation analysis results between microarray and qRT-RCR experiments based on 15 selected genes representing the OsDREB1 regulon and candidate genes of introgressed segments. Gene expression values were transformed to a log2 scale. The microarray data log2-value (X-axis) were plotted against the qRT-PCR log2-value (Y-axis). [file 1471-2164-13-461-S6.ppt]

## Slide 1
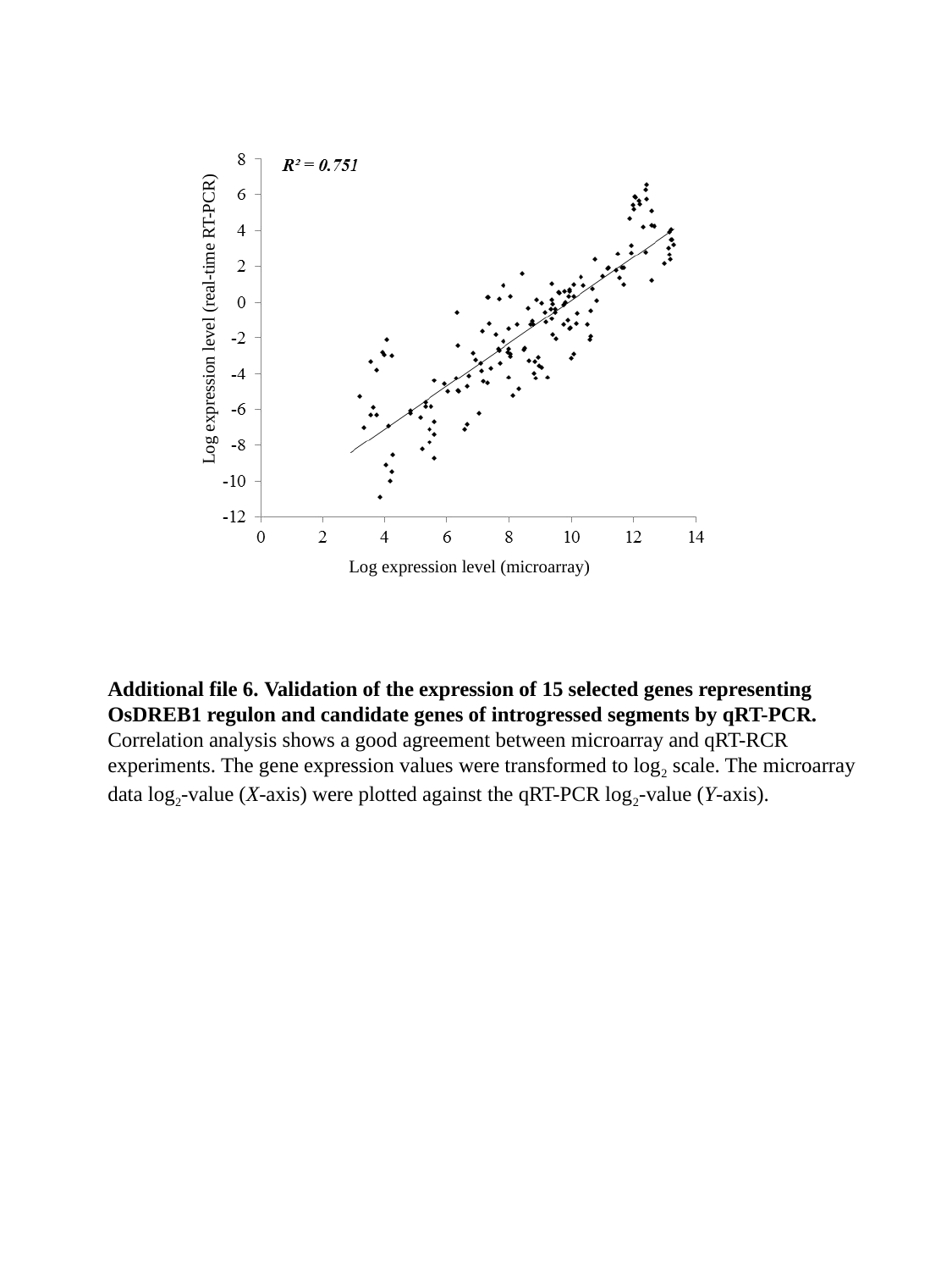

Log expression level (real-time RT-PCR)
Log expression level (microarray)
Additional file 6. Validation of the expression of 15 selected genes representing OsDREB1 regulon and candidate genes of introgressed segments by qRT-PCR. Correlation analysis shows a good agreement between microarray and qRT-RCR experiments. The gene expression values were transformed to log2 scale. The microarray data log2-value (X-axis) were plotted against the qRT-PCR log2-value (Y-axis).
