# Supplementary material for: Genome-wide gene expression profiling of introgressed indica rice alleles associated with seedling cold tolerance improvement in a japonica rice background
Source: BMC Genomics. 2012 Sep 7;13:461. doi: 10.1186/z (PMC3526417; doi:10.1186/z)
Supplement: Additional file 7 — Comparative diagram of the total number of differentially regulated genes between C418 and K354 under cold stress. A PowerPoint file containing (A) Cold-induced genes; (B) Cold-repressed genes. Blue, red, and green bars indicate K354-specific (blue bar), common (red bar), and C418-specific (green bar) regulated genes by cold stress at phase I (2–6 h), phase II (12 h), and phase III (24–48 h) of cold-response, respectively. [file 1471-2164-13-461-S7.ppt]

## Slide 1
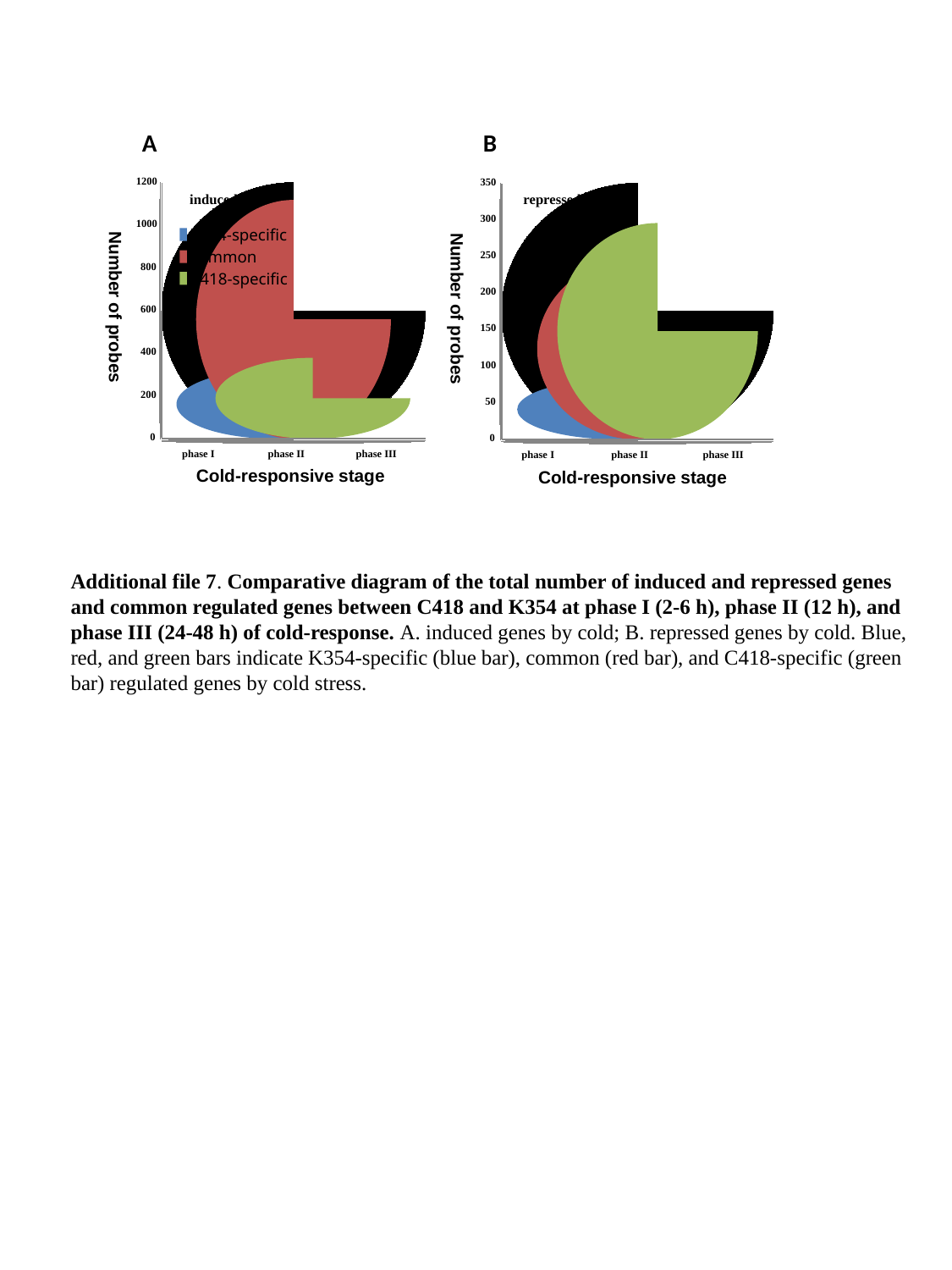

A
B
1200
Induced
1000
800
600
400
200
0
phase I
phase II
phase III
350
300
250
200
150
100
50
0
phase I
phase II
phase III
induced
repressed
K354-specific
Number of probes
Number of probes
Common
C418-specific
Cold-responsive stage
Cold-responsive stage
Additional file 7. Comparative diagram of the total number of induced and repressed genes and common regulated genes between C418 and K354 at phase I (2-6 h), phase II (12 h), and phase III (24-48 h) of cold-response. A. induced genes by cold; B. repressed genes by cold. Blue, red, and green bars indicate K354-specific (blue bar), common (red bar), and C418-specific (green bar) regulated genes by cold stress.
