# Supplementary material for: Genome-wide gene expression profiling of introgressed indica rice alleles associated with seedling cold tolerance improvement in a japonica rice background
Source: BMC Genomics. 2012 Sep 7;13:461. doi: 10.1186/z (PMC3526417; doi:10.1186/z)
Supplement: Additional file 8 — Common genes regulated by cold in both genotypes at all cold-responsive stages. Word file containing 130 and 21 genes commonly induced and repressed by cold in both genotypes at all cold-responsive stages. [file 1471-2164-13-461-S8.doc]

**Additional file 8.** 130 and 21 genes commonly induced and repressed by cold in both genotypes at all cold-responsive stages.

| **Probe ID** | **Locus ID** | **K354 stress/control** | | | | | **C418 stress/control** | | | | | **Annotation** |
| --- | --- | --- | --- | --- | --- | --- | --- | --- | --- | --- | --- | --- |
|  |  | **2h** | **6h** | **12h** | **24h** | **48h** | **2h** | **6h** | **12h** | **24h** | **48h** |  |
| **Induced by cold** |  |  |  |  |  |  |  |  |  |  |  |  |
| Os.46857.1.S2_at | LOC_Os10g42770 | 6.63 | 9.88 | 16.12 | 12.67 | 41.17 | 7.45 | 13.86 | 16.47 | 18.03 | 39.64 | a_IG002N01.7, putative |
| Os.54944.1.S1_at | LOC_Os02g52670 | . | 11.08 | 31.00 | 27.34 | 36.16 | 9.31 | 17.14 | 26.15 | 60.59 | 67.32 | AP2 domain containing protein, expressed(AP2/EREBP family) |
| Os.8031.1.S1_at | LOC_Os01g21120 | . | 7.32 | 14.76 | 30.22 | 40.71 | 6.16 | 11.86 | 14.62 | 41.66 | 72.84 | AP2 domain containing protein, expressed(AP2/EREBP family) |
| Os.46602.1.S1_at | LOC_Os10g41330 | 5.11 | 12.50 | 31.32 | 28.42 | 49.83 | 6.10 | 28.49 | 70.11 | 54.44 | 94.04 | AP2 domain containing protein, expressed(AP2/EREBP family) |
| OsAffx.17366.1.S1_at | LOC_Os08g36920 | 11.40 | 48.74 | 79.05 | 87.35 | 106.83 | 95.02 | 111.47 | 96.09 | 204.63 | 165.23 | AP2 domain containing protein, expressed(AP2/EREBP family) |
| Os.52451.1.A1_at | LOC_Os09g28440 | 28.15 | 117.45 | 122.04 | 156.74 | 204.08 | 89.59 | 95.22 | 95.41 | 197.25 | 194.38 | AP2 domain containing protein, expressed(AP2/EREBP family) |
| Os.10884.1.S1_at | LOC_Os03g58790 | . | 6.16 | 8.15 | 13.92 | 17.81 | 5.00 | 7.09 | 11.87 | 23.14 | 31.26 | ATPase, putative, expressed |
| OsAffx.3295.1.S1_at | LOC_Os03g22270 | 10.82 | 38.41 | 52.90 | 80.64 | 107.08 | 8.44 | 42.29 | 71.29 | 101.61 | 109.42 | auxin-repressed protein, putative, expressed |
| Os.51129.1.S1_at | LOC_Os06g39590 | . | 22.08 | 44.69 | 53.07 | 76.46 | . | 19.67 | 35.19 | 47.24 | 80.37 | Auxin-responsive protein IAA23(AUX/IAA family ) |
| Os.46618.1.S1_at | LOC_Os10g41550 | . | 5.54 | 9.15 | 14.39 | 19.92 | . | 6.32 | 11.96 | 19.61 | 26.49 | beta-amylase, putative, expressed |
| Os.46832.1.S1_at | LOC_Os10g28240 | . | 25.45 | 54.94 | 67.33 | 94.94 | 8.35 | 25.49 | 47.42 | 74.39 | 119.76 | calcium-transporting ATPase, plasma membrane-type, putative, expressed |
| OsAffx.12442.1.S1_at | LOC_Os02g41580 | . | 5.13 | 12.69 | 18.68 | 23.58 | . | 6.90 | 12.75 | 22.63 | 33.91 | CAMK_CAMK_like.14 - CAMK includes calcium/calmodulin depedent protein kinases, expressed |
| Os.46328.1.S1_at | LOC_Os04g46980 | . | 17.83 | 26.12 | 20.58 | 24.59 | 12.90 | 15.60 | 28.31 | 27.29 | 22.89 | cis-zeatin O-glucosyltransferase, putative, expressed |
| Os.49999.1.S1_x_at | LOC_Os03g18910 | . | 7.52 | 13.43 | 10.45 | 16.42 | 10.14 | 9.45 | 11.83 | 14.35 | 17.11 | COBRA-like protein 7 precursor, putative, expressed |
| OsAffx.18426.1.S1_x_at | LOC_Os10g28170 | . | 17.85 | 25.63 | 18.81 | 25.75 | 8.34 | 22.84 | 23.60 | 38.83 | 36.74 | conserved hypothetical protein |
| Os.9445.1.S1_at | Os04g0635400 | 13.40 | 75.44 | 64.90 | 29.73 | 18.89 | 9.78 | 42.61 | 45.77 | 32.69 | 15.47 | Conserved hypothetical protein. |
| Os.38109.1.S1_at | Os01g0668700 | . | 5.17 | 17.56 | 30.06 | . | . | 5.80 | 14.78 | 26.01 | 52.14 | Conserved hypothetical protein. |
| Os.6092.1.S1_at | LOC_Os02g44230 | . | 22.19 | 40.15 | 40.57 | 48.14 | 24.09 | 46.40 | 55.93 | 56.86 | 53.71 | CPuORF22 - conserved peptide uORF-containing transcript, expressed |
| Os.17019.1.A1_at | LOC_Os02g21920 | . | 7.81 | 10.48 | 13.82 | 18.78 | . | 7.46 | 9.83 | 11.21 | 14.50 | CPuORF24 - conserved peptide uORF-containing transcript, expressed |
| Os.4786.1.S2_at | LOC_Os04g42090 | 5.04 | 6.67 | 11.36 | 21.67 | 37.76 | . | 7.10 | 16.85 | 22.28 | 35.82 | CPuORF7 - conserved peptide uORF-containing transcript, expressed |
| OsAffx.15997.1.S1_at | LOC_Os06g49470 | . | 5.33 | 12.96 | 15.26 | 38.80 | . | 9.08 | 13.96 | 22.09 | 41.64 | Cyclophilin |
| OsAffx.32224.1.A1_at | LOC_Os06g46434 | . | 10.51 | 13.25 | 6.72 | 6.24 | . | 9.44 | 10.97 | . | 4.30 | cytochrome c biogenesis protein ccsA, putative |
| Os.12145.1.S1_at | LOC_Os02g47470 | 8.45 | 11.41 | 13.63 | 13.03 | 13.21 | 33.76 | 34.35 | 36.28 | 38.01 | 34.64 | cytochrome P450, putative, expressed |
| Os.14105.1.S1_at | LOC_Os11g05380 | . | 18.06 | 32.93 | 44.97 | 55.42 | . | 47.58 | 44.52 | 114.11 | 127.44 | cytochrome P450, putative, expressed |
| Os.14125.1.S1_at | LOC_Os09g35030 | 11.87 | 15.87 | 15.64 | 10.98 | 12.35 | 20.95 | 24.41 | 18.97 | 20.65 | 18.31 | Dehydration-responsive element-binding protein 1A(AP2/EREBP family) |
| Os.5816.1.S1_at | LOC_Os09g35010 | 15.01 | 19.95 | 21.29 | 20.18 | 19.03 | 24.42 | 28.46 | 29.37 | 29.60 | 26.36 | Dehydration-responsive element-binding protein 1B(AP2/EREBP family) |
| OsAffx.27442.1.S1_at | LOC_Os06g03670 | 6.08 | 35.80 | 50.34 | 32.82 | 49.97 | 33.09 | 60.14 | 58.13 | 65.41 | 51.86 | Dehydration-responsive element-binding protein 1C(AP2/EREBP family) |
| Os.40428.1.S1_at | LOC_Os01g73770 | . | 16.29 | 20.98 | 12.17 | 12.87 | . | 26.25 | 9.73 | 31.07 | 7.72 | Dehydration-responsive element-binding protein 1F(AP2/EREBP family) |
| Os.51078.1.S1_at | LOC_Os02g45450 | . | 7.32 | 10.40 | 8.21 | 12.05 | 24.22 | 35.24 | 40.12 | 41.80 | 41.95 | Dehydration-responsive element-binding protein 1G(AP2/EREBP family) |
| Os.27915.1.S1_at | LOC_Os02g04130 | 6.89 | 16.56 | 19.70 | 23.95 | 42.12 | 13.08 | 15.53 | 14.94 | 23.44 | 42.05 | DUF1645 domain containing protein, putative, expressed |
| Os.19385.1.S1_at | LOC_Os01g52730 | 6.27 | 8.36 | 9.58 | 8.32 | 8.98 | 9.59 | 7.32 | 6.58 | 9.46 | 9.32 | DUF584 domain containing protein, putative, expressed |
| Os.11719.1.S2_at | LOC_Os07g46670 | . | 19.32 | 40.41 | 52.53 | 56.52 | 5.42 | 33.06 | 51.86 | 74.19 | 67.11 | early response to dehydration 15, putative, expressed |
| Os.27569.1.S2_at | LOC_Os09g30490 | . | 5.94 | 14.26 | 21.06 | 35.80 | . | 9.06 | 16.53 | 29.21 | 48.16 | EF hand family protein, expressed |
| Os.3766.1.S1_at | LOC_Os01g58420 | 6.37 | 7.84 | 8.57 | 6.70 | 9.42 | 7.81 | 6.74 | 6.18 | 6.81 | 7.07 | Ethylene responsive element binding factor3(AP2/EREBP family) |
| Os.53052.1.S1_at | LOC_Os01g67820 | . | 6.50 | 14.07 | 21.10 | 37.64 | 8.49 | 9.43 | 14.52 | 20.96 | 31.31 | exo70 exocyst complex subunit domain containing protein, expressed |
| Os.38806.1.A1_x_at | LOC_Os06g04230 | . | 9.03 | 8.04 | 8.09 | 7.88 | 6.32 | 8.01 | 8.65 | 8.85 | 7.73 | expressed protein |
| OsAffx.31986.1.S1_at | LOC_Os12g32610 | . | 9.62 | 11.56 | 8.71 | 9.83 | 11.92 | 9.05 | 9.27 | 9.59 | 7.75 | expressed protein |
| OsAffx.4280.2.A1_at | LOC_Os05g08910 | . | 13.20 | 15.56 | 6.11 | 8.95 | 6.22 | 8.09 | 9.15 | 8.64 | 12.22 | expressed protein |
| Os.606.1.S1_at | LOC_Os06g04220 | . | 8.88 | 12.48 | 9.79 | 8.00 | 5.43 | 8.53 | 11.99 | 12.22 | 12.54 | expressed protein |
| Os.57301.1.S1_at | LOC_Os02g52170 | . | 7.06 | 8.18 | 7.14 | 12.59 | 10.38 | 8.74 | 8.73 | 9.36 | 14.02 | expressed protein |
| Os.14393.2.S1_at | LOC_Os09g24970 | . | 6.36 | 14.15 | 12.26 | 19.29 | . | 9.04 | 13.42 | 16.47 | 19.80 | expressed protein |
| Os.53581.1.S1_at | LOC_Os09g37080 | . | 5.93 | 8.80 | 7.85 | 15.61 | 11.85 | 6.33 | 5.71 | 12.57 | 22.68 | expressed protein |
| Os.2426.1.A1_at | LOC_Os11g10470 | . | 8.63 | 10.63 | 8.72 | 12.24 | 8.51 | 12.41 | 14.20 | 14.74 | 24.56 | expressed protein |
| Os.52280.1.S1_at | LOC_Os06g46140 | . | 7.85 | 11.62 | 20.85 | 20.30 | 6.47 | 9.73 | 15.34 | 23.10 | 28.50 | expressed protein |
| Os.27807.1.S1_a_at | LOC_Os03g01740 | . | 7.93 | 9.33 | 14.95 | 19.17 | 9.86 | 10.60 | 10.14 | 18.56 | 28.86 | expressed protein |
| Os.5213.1.S1_at | LOC_Os02g44720 | 8.44 | 19.80 | 18.77 | 14.01 | 20.67 | 18.37 | 25.98 | 18.97 | 27.60 | 29.65 | expressed protein |
| Os.55519.1.S1_at | LOC_Os02g09990 | . | 9.34 | 8.11 | . | 12.29 | 23.53 | 8.45 | 10.81 | 13.08 | 33.23 | expressed protein |
| Os.14210.1.S1_at | LOC_Os10g36360 | . | 7.07 | 10.35 | 19.04 | 23.69 | . | 11.36 | 17.20 | 36.38 | 38.66 | expressed protein |
| Os.56018.1.S1_at | LOC_Os05g44060 | . | 15.63 | 31.52 | 24.84 | 39.56 | 14.10 | 21.56 | 22.89 | 42.08 | 39.43 | expressed protein |
| Os.55394.1.S1_at | LOC_Os03g52410 | . | 19.68 | 28.90 | 24.23 | 34.45 | 5.95 | 18.93 | 22.12 | 36.88 | 40.07 | expressed protein |
| Os.55850.1.S1_at | LOC_Os08g34800 | . | 5.92 | 10.22 | 18.92 | 27.77 | . | 5.95 | 9.82 | 23.54 | 40.95 | expressed protein |
| Os.8774.1.S1_at | LOC_Os05g37520 | . | 6.86 | 16.39 | 21.79 | 43.18 | 6.60 | 10.83 | 15.92 | 29.43 | 48.87 | expressed protein |
| Os.23186.1.S1_at | LOC_Os05g04000 | . | 9.28 | 24.61 | 26.55 | 48.76 | . | 14.41 | 27.55 | 37.78 | 55.84 | expressed protein |
| Os.50345.1.S1_at | LOC_Os01g07364 | . | 10.84 | 18.14 | 47.83 | 73.03 | . | 20.43 | 34.48 | 75.08 | 144.74 | expressed protein |
| OsAffx.6035.1.S1_at | LOC_Os08g35580 | . | 8.91 | 12.33 | 5.66 | . | 12.13 | 9.55 | 9.00 | 7.27 | . | expressed protein |
| Os.52733.1.S1_at | LOC_Os04g03980 | . | 5.46 | 13.55 | 25.13 | 35.77 | . | 5.62 | 17.10 | 34.52 | 48.97 | flavin monooxygenase, putative, expressed |
| Os.47946.1.S1_at | LOC_Os07g33910 | . | 9.81 | 30.21 | . | 37.01 | . | 14.69 | 13.42 | 17.49 | 40.71 | Glucose-6-phosphate/phosphate-translocator precursor |
| Os.53972.1.S1_at | LOC_Os04g46970 | . | 12.16 | 41.45 | 75.45 | 115.25 | 11.69 | 23.70 | 69.33 | 100.33 | 136.81 | glucosyltransferase, putative, expressed |
| Os.52150.1.S1_at | LOC_Os04g45970 | 5.37 | 14.12 | 14.28 | 32.29 | 19.41 | . | 11.57 | 15.94 | 25.42 | 24.95 | glutamate dehydrogenase protein, putative, expressed |
| Os.55382.1.S1_at | Os03g0188500 | . | 11.01 | 12.47 | 15.09 | 17.70 | 8.52 | 14.70 | 17.91 | 20.48 | 24.97 | Glutelin family protein |
| Os.28030.1.S1_s_at | LOC_Os06g48160 | . | 5.98 | 13.69 | 45.52 | 58.39 | . | 10.62 | 18.60 | 62.78 | 91.98 | glycosyl hydrolases family 16, putative, expressed |
| Os.26079.1.S1_at | LOC_Os02g50600 | . | 8.84 | 12.70 | 12.85 | 20.17 | 8.09 | 7.47 | 8.33 | 14.37 | 22.77 | glycosyl transferase 8 domain containing protein, putative, expressed |
| Os.35024.1.S1_at | LOC_Os01g31370 | . | 5.68 | 12.05 | 13.94 | 46.24 | 8.36 | 10.86 | 17.16 | 28.73 | 64.27 | glycosyltransferase, putative, expressed |
| Os.12119.1.S1_at | LOC_Os01g64470 | . | 5.27 | 6.61 | 5.01 | 5.02 | 8.31 | 6.40 | 7.11 | 7.08 | 7.43 | harpin-induced protein 1 domain containing protein, expressed |
| Os.23778.1.S1_at | LOC_Os02g32590 | . | 8.53 | 20.66 | 17.97 | 39.58 | 7.06 | 10.89 | 10.21 | 19.56 | 42.41 | Heat stress transcription factor A-3(HSF family ) |
| Os.49591.1.S1_at | LOC_Os03g05750 | 6.70 | 17.17 | 22.25 | 21.60 | 28.19 | 19.69 | 14.73 | 16.28 | 20.02 | 21.61 | heavy-metal-associated domain-containing protein, putative, expressed |
| Os.6321.1.S1_at | LOC_Os01g63690 | 5.10 | 7.97 | 8.47 | 8.93 | 10.07 | 7.43 | 8.39 | 8.65 | 9.79 | 10.15 | hs1, putative, expressed |
| Os.20548.1.S1_at | LOC_Os04g51680 | . | 5.33 | 7.14 | 9.54 | 8.55 | 5.33 | 6.13 | 7.96 | 11.48 | 9.89 | hypothetical protein |
| Os.49555.1.S1_at | LOC_Os02g56250 | . | 6.91 | 10.15 | 8.15 | 13.46 | 7.33 | 8.53 | 10.58 | 10.16 | 13.01 | Hypothetical protein |
| OsAffx.5608.1.S1_at | LOC_Os07g42324 | . | 8.20 | 35.29 | 34.18 | 34.97 | . | 12.74 | 28.51 | 33.43 | 47.19 | hypothetical protein |
| Os.53517.1.S1_at | Os12g0136300 | . | 5.74 | 17.28 | 100.91 | 119.30 | . | 11.38 | 28.37 | 88.31 | 120.02 | Hypothetical protein |
| Os.16062.1.S1_at | LOC_Os10g22980 | . | 7.60 | 14.48 | 9.31 | 12.50 | . | 7.06 | 13.08 | 10.72 | 10.83 | Leucine Rich Repeat family protein, expressed |
| Os.10091.1.S1_at | LOC_Os03g19070 | 15.11 | 68.69 | 72.41 | 43.91 | 72.41 | 30.84 | 71.54 | 66.37 | 61.98 | 83.54 | long cell-linked locus protein, putative, expressed |
| OsAffx.14324.1.S1_at | LOC_Os04g48290 | . | 6.53 | 13.25 | 7.79 | 14.17 | 26.83 | 18.20 | 17.21 | 21.21 | 25.05 | MATE efflux family protein, putative, expressed |
| Os.8149.1.S1_at | LOC_Os02g46030 | 7.42 | 7.62 | 10.09 | 10.50 | 13.65 | 10.64 | 10.34 | 16.01 | 15.83 | 19.39 | MYB family transcription factor, putative, expressed(MYB family ) |
| Os.10172.1.S1_at | LOC_Os02g41510 | . | 7.12 | 19.13 | 12.20 | 18.16 | 8.30 | 9.77 | 14.42 | 20.45 | 19.67 | MYB family transcription factor, putative, expressed(MYB family ) |
| Os.14823.1.S1_s_at | LOC_Os03g20090 | 7.00 | 17.86 | 29.05 | 23.30 | 28.87 | 16.88 | 17.23 | 19.08 | 24.56 | 25.33 | MYB family transcription factor, putative, expressed(MYB family ) |
| Os.3388.2.S1_a_at | LOC_Os04g43680 | . | 10.45 | 15.21 | 18.92 | 19.61 | 6.43 | 17.83 | 19.43 | 25.97 | 26.45 | MYB family transcription factor, putative, expressed(MYB family ) |
| Os.5335.1.S1_at | LOC_Os04g49450 | 5.93 | 24.31 | 44.89 | 64.28 | 81.36 | 19.56 | 31.64 | 53.46 | 71.49 | 85.68 | MYB family transcription factor, putative, expressed(MYB family ) |
| Os.49746.1.S1_at | LOC_Os01g74020 | . | 5.79 | 14.45 | 51.98 | 126.71 | . | 6.65 | 12.93 | 47.27 | 110.87 | MYB family transcription factor, putative, expressed(GARP-G2-like family ) |
| Os.5549.1.S1_at | LOC_Os07g12340 | . | 9.95 | 10.24 | 10.21 | 10.70 | . | 7.32 | 7.75 | 8.45 | 9.76 | NAC domain-containing protein 67, putative, expressed(NAC family ) |
| OsAffx.20471.1.S1_x_at | LOC_Os04g30930 | . | 7.52 | 10.82 | 10.85 | 15.70 | . | 6.84 | 12.03 | 10.79 | 10.59 | NB-ARC domain containing protein |
| Os.24338.1.A1_at | LOC_Os01g68020 | 6.11 | 9.50 | 11.45 | 9.43 | 13.12 | . | 5.31 | 5.60 | 5.16 | 6.10 | no hits found |
| OsAffx.32295.1.S1_at | BA000029 | . | 8.67 | 11.12 | 10.37 | 12.67 | . | 5.17 | 8.72 | 7.84 | 9.56 | no hits found |
| Os.53459.1.S1_at | AK072753 | . | 7.97 | 15.30 | 19.13 | 22.61 | 5.13 | . | 5.65 | 11.09 | 13.94 | no hits found |
| OsAffx.9069.1.S1_x_at | NM_193114 | . | 9.99 | 8.86 | 19.26 | 24.06 | . | 7.27 | 10.22 | 14.02 | 21.97 | no hits found |
| Os.57147.1.S1_at | LOC_Os03g09230 | . | 8.18 | 10.49 | 17.04 | 17.79 | . | 6.71 | 16.10 | 15.56 | 16.49 | Non-specific lipid-transfer protein |
| Os.47407.1.A1_at | LOC_Os01g72100 | . | 5.42 | 8.48 | . | 9.72 | . | 5.79 | 10.12 | 8.74 | 6.48 | OsCML10 - Calmodulin-related calcium sensor protein, expressed |
| Os.10058.1.S1_at | LOC_Os05g31620 | 6.92 | 9.40 | 9.54 | 10.42 | 12.99 | 11.39 | 7.48 | 8.04 | 9.83 | 10.93 | OsCML15 - Calmodulin-related calcium sensor protein, expressed |
| Os.16401.1.S1_at | LOC_Os03g21380 | . | 8.40 | 9.35 | 7.71 | 8.29 | 6.62 | 9.45 | 9.27 | 8.82 | 9.09 | OsCML27 - Calmodulin-related calcium sensor protein, expressed |
| Os.18922.2.S1_at | LOC_Os01g72530 | . | 7.59 | 7.51 | 7.01 | 9.58 | 7.50 | 6.68 | 6.68 | 7.07 | 8.58 | OsCML31 - Calmodulin-related calcium sensor protein, expressed |
| Os.11125.1.S1_a_at | LOC_Os01g59690 | . | 6.23 | 9.09 | 10.65 | 11.95 | . | 5.12 | 6.99 | 9.98 | 9.38 | OsFBX28 - F-box domain containing protein, expressed |
| Os.53268.1.S1_at | LOC_Os09g17152 | . | 7.79 | 16.60 | 22.44 | 25.77 | . | 6.54 | 8.33 | 23.62 | 25.47 | OsFBX319 - F-box domain containing protein, expressed |
| Os.25109.1.S1_at | LOC_Os11g02600 | . | 9.78 | 12.01 | 13.72 | 33.89 | 7.55 | 11.52 | 13.91 | 17.45 | 33.29 | OsMan08 - Endo-Beta-Mannanase, expressed |
| Os.37067.1.S1_at | LOC_Os10g37760 | . | 15.74 | 33.80 | 25.65 | 46.19 | 26.06 | 23.56 | 32.51 | 40.06 | 56.99 | OsRhmbd17 - Putative Rhomboid homologue, expressed |
| Os.51732.1.S1_at | LOC_Os09g31438 | . | 8.52 | 16.00 | 12.05 | 7.41 | . | 15.20 | 15.56 | 17.35 | 11.15 | OsSPL17 - SBP-box gene family member, expressed |
| Os.30568.1.S1_at | LOC_Os01g60640 | . | 7.25 | 10.49 | 13.93 | 13.60 | 7.50 | 6.42 | 7.45 | 15.21 | 11.78 | OsWRKY21 - Superfamily of TFs having WRKY and zinc finger domains, expressed(WRKY family ) |
| Os.9194.1.S1_at | LOC_Os02g52040 | . | 7.44 | 8.76 | 14.27 | 14.66 | 10.42 | 15.11 | 15.65 | 22.61 | 19.21 | phosphate-induced protein 1 conserved region domain containing protein, expressed |
| Os.7228.1.S1_at | LOC_Os02g51970 | . | 9.52 | 14.34 | 57.03 | 34.40 | . | 11.09 | 15.99 | 40.71 | 44.81 | phosphate-induced protein 1 conserved region domain containing protein, expressed |
| Os.12240.1.S1_at | LOC_Os02g52010 | . | 23.20 | 34.88 | 37.84 | 46.00 | 9.16 | 28.12 | 31.93 | 62.00 | 85.36 | phosphate-induced protein 1 conserved region domain containing protein, expressed |
| Os.13835.2.S2_at | LOC_Os01g51920 | 5.03 | 21.35 | 33.92 | 24.11 | 27.36 | 7.99 | 15.91 | 19.36 | 27.74 | 25.42 | phosphotransferase, putative, expressed |
| Os.49534.1.S1_x_at | LOC_Os08g17160 | 9.11 | 47.63 | 73.74 | 88.75 | 68.71 | 11.82 | 36.71 | 54.63 | 133.93 | 109.25 | plastocyanin-like domain containing protein, putative, expressed |
| OsAffx.31541.1.S1_s_at | LOC_Os11g01610 | . | 6.23 | 9.54 | 18.65 | 26.78 | . | 6.29 | 10.60 | 18.42 | 32.62 | prenylated rab acceptor, putative, expressed |
| OsAffx.12910.1.S1_at | LOC_Os03g18150 | . | 5.43 | 7.17 | 7.31 | 10.03 | 6.14 | 5.40 | 6.94 | 8.43 | 10.14 | protein phosphatase 2C, putative, expressed |
| Os.51297.1.S1_at | LOC_Os02g35080 | 8.70 | 13.92 | 42.49 | 28.40 | 27.59 | . | 15.91 | 40.60 | 35.40 | 48.29 | Putative T-complex protein 11 |
| Os.46518.1.S1_at | LOC_Os10g35640 | . | 26.88 | 58.58 | 69.96 | 95.53 | . | 9.81 | 17.56 | 32.03 | 43.87 | Rf1, mitochondrial precursor, putative, expressed |
| Os.30910.1.S1_at | LOC_Os01g73630 | . | 6.89 | 9.67 | 9.85 | 9.39 | . | 8.02 | 10.50 | 10.85 | 10.51 | rhoGAP domain containing protein, expressed |
| Os.54700.1.S1_at | LOC_Os03g55560 | . | 5.57 | 7.91 | 5.28 | 6.99 | . | 6.60 | 7.70 | 6.14 | 6.78 | STE_MEKK_ste11_MAP3K.15 - STE kinases include homologs to sterile 7, sterile 11 and sterile 20 from yeast, expressed |
| Os.6085.1.S1_at | LOC_Os05g46760 | . | 15.53 | 42.19 | 49.10 | 80.84 | 7.97 | 12.43 | 22.76 | 50.68 | 96.79 | STE_MEKK_ste11_MAP3K.19 - STE kinases include homologs to sterile 7, sterile 11 and sterile 20 from yeast, expressed |
| Os.5940.1.S1_at | LOC_Os01g50370 | . | 6.64 | 14.27 | 14.86 | 25.71 | . | 7.37 | 9.48 | 21.09 | 28.76 | STE_MEKK_ste11_MAP3K.4 - STE kinases include homologs to sterile 7, sterile 11 and sterile 20 from yeast, expressed |
| Os.5044.1.S1_at | LOC_Os01g50410 | . | 6.10 | 11.81 | 12.87 | 12.19 | . | 13.32 | 11.36 | 28.18 | 27.88 | STE_MEKK_ste11_MAP3K.6 - STE kinases include homologs to sterile 7, sterile 11 and sterile 20 from yeast, expressed |
| Os.32366.1.S1_at | LOC_Os01g50420 | . | 14.91 | 27.13 | 31.06 | 29.98 | 5.98 | 23.56 | 28.67 | 50.10 | 48.44 | STE_MEKK_ste11_MAP3K.7 - STE kinases include homologs to sterile 7, sterile 11 and sterile 20 from yeast, expressed |
| Os.46793.1.S1_at | LOC_Os10g05660 | . | 6.24 | 8.54 | 9.96 | 5.30 | . | 5.88 | 6.78 | 8.21 | 6.70 | thaumatin, putative, expressed |
| OsAffx.24166.1.S1_at | LOC_Os02g08440 | . | 7.54 | 14.62 | 19.49 | 23.70 | 7.47 | 11.31 | 17.81 | 31.48 | 31.15 | Transcription factor OsWRKY71(WRKY family ) |
| Os.53121.1.S1_at | LOC_Os07g08060 | . | 5.29 | 7.60 | 16.53 | 55.66 | . | 6.80 | 9.73 | 22.86 | 66.17 | transmembrane BAX inhibitor motif-containing protein, putative, expressed |
| Os.3808.4.S1_x_at | LOC_Os01g09220 | 7.11 | 10.22 | 10.97 | 11.99 | 12.92 | 11.22 | 11.52 | 11.86 | 13.43 | 14.41 | transposon protein, putative, CACTA, En/Spm sub-class, expressed |
| Os.26884.1.S1_a_at | LOC_Os01g67810 | . | 7.07 | 11.68 | 12.10 | 17.78 | 10.04 | 10.65 | 14.04 | 17.71 | 19.08 | transposon protein, putative, unclassified, expressed |
| Os.8158.1.S1_at | LOC_Os02g54890 | . | 6.08 | 7.06 | 6.30 | 8.37 | 11.32 | 6.18 | 5.35 | 8.30 | 9.53 | UDP-glucuronate 4-epimerase, putative, expressed |
| Os.8859.1.S1_s_at | LOC_Os07g07990 | . | 5.17 | 6.74 | 13.72 | 23.11 | . | 6.14 | 8.19 | 14.17 | 30.13 | uncharacterized protein At4g06744 precursor, putative, expressed |
| Os.51226.1.S1_at | LOC_Os03g52910 | . | 11.78 | 24.52 | 27.87 | 43.61 | . | 8.42 | 12.44 | 17.74 | 30.72 | uncharacterized UPF0114 domain containing protein, expressed |
| OsAffx.22380.1.S1_at | LOC_Os07g39680 | 8.82 | 20.17 | 12.53 | 6.92 | 10.31 | 10.68 | 12.19 | 7.39 | 7.49 | 8.47 | UV-induced protein uvi15, putative, expressed |
| Os.9913.1.S1_at | LOC_Os04g54230 | 10.72 | 17.17 | 12.39 | 5.03 | 8.04 | 8.90 | 9.08 | 6.50 | . | 4.59 | wound induced protein, putative, expressed |
| OsAffx.14412.2.S1_s_at | LOC_Os04g54240 | 11.60 | 24.67 | 19.97 | 8.31 | 6.52 | 10.63 | 13.54 | 9.91 | 9.91 | 5.74 | wound induced protein, putative, expressed |
| Os.37565.1.S1_at | LOC_Os05g25770 | . | 11.62 | 19.09 | 26.65 | 34.74 | 6.86 | 16.38 | 23.42 | 36.22 | 51.82 | WRKY-type transcription factor 45-1(WRKY family ) |
| Os.46849.1.S1_at | LOC_Os10g25230 | . | 13.62 | 18.72 | 17.53 | 21.59 | 6.77 | 29.71 | 22.72 | 39.06 | 35.34 | ZIM domain containing protein, putative, expressed(ZIM family ) |
| Os.8088.1.S1_at | LOC_Os03g08310 | . | 19.67 | 37.12 | 39.70 | 40.60 | 5.69 | 36.86 | 37.47 | 65.54 | 62.55 | ZIM motif family protein, expressed(ZIM family ) |
| Os.9923.1.S1_at | LOC_Os03g08330 | . | 9.62 | 16.87 | 16.81 | 16.83 | . | 17.65 | 20.74 | 32.13 | 30.47 | ZIM motif family protein, expressed (ZIM family ) |
| Os.38236.1.S1_at | LOC_Os01g74040 | . | 8.87 | 11.24 | 9.17 | 10.93 | 13.20 | 7.86 | 7.82 | 8.51 | 8.89 | zinc finger, RING-type, putative, expressed |
| Os.28441.1.S1_at | LOC_Os03g60560 | . | 11.39 | 17.37 | 13.67 | 30.79 | 9.15 | 9.12 | 10.68 | 13.85 | 29.89 | ZOS3-21 - C2H2 zinc finger protein, expressed(C2H2 family ) |
| Os.15874.1.A1_at | LOC_Os03g60570 | 7.35 | 27.78 | 41.83 | 32.15 | 51.66 | 34.23 | 30.88 | 39.16 | 49.09 | 61.78 | ZOS3-22 - C2H2 zinc finger protein, expressed(C2H2 family ) |
| **Repressed by cold** |  |  |  |  |  |  |  |  |  |  |  |  |
| Os.9950.1.S1_at | LOC_Os06g14240 | 0.12 | 0.08 | 0.05 | 0.04 | 0.04 | 0.08 | 0.05 | 0.03 | 0.02 | 0.02 | hsp20/alpha crystallin family protein, putative, expressed |
| Os.12257.1.S1_at | LOC_Os02g52150 | 0.06 | 0.06 | 0.06 | 0.08 | 0.17 | 0.05 | 0.04 | 0.04 | 0.04 | 0.12 | heat shock 22 kDa protein, mitochondrial precursor, putative, expressed |
| Os.49151.1.S1_at | LOC_Os11g44800 | . | 0.09 | 0.06 | 0.09 | 0.08 | . | 0.10 | 0.07 | 0.05 | 0.04 | expressed protein |
| Os.175.1.S1_at | LOC_Os04g59440 | . | 0.12 | 0.11 | 0.07 | 0.06 | . | 0.12 | 0.08 | 0.05 | 0.05 | chlorophyll A-B binding protein, putative, expressed |
| Os.37713.1.S1_at | LOC_Os07g38960 | 0.18 | 0.11 | 0.07 | 0.03 | 0.04 | . | 0.09 | 0.06 | 0.05 | 0.03 | chlorophyll A-B binding protein, putative, expressed |
| Os.5698.1.S1_s_at | LOC_Os05g33700 | 0.09 | 0.07 | 0.05 | 0.07 | 0.07 | 0.07 | 0.05 | 0.05 | 0.06 | 0.07 | 4F5 protein family protein |
| Os.52495.1.S1_at | LOC_Os03g07410 | . | 0.11 | 0.09 | 0.07 | 0.11 | . | 0.12 | 0.06 | 0.07 | 0.07 | expressed protein |
| Os.51629.1.S1_at | LOC_Os02g09850 | . | 0.11 | 0.08 | 0.15 | 0.08 | 0.18 | 0.10 | 0.10 | 0.08 | 0.08 | expressed protein |
| Os.46835.1.S1_at | LOC_Os10g21860 | . | 0.18 | 0.13 | 0.09 | . | . | 0.18 | 0.10 | 0.09 | 0.28 | expressed protein |
| Os.54900.1.S1_at | LOC_Os01g66990 | . | 0.12 | 0.12 | 0.09 | 0.10 | . | 0.14 | 0.11 | 0.10 | 0.09 | hypothetical protein |
| Os.11941.2.S1_at | LOC_Os09g35790 | 0.20 | 0.19 | 0.16 | 0.17 | 0.11 | 0.15 | 0.13 | 0.13 | 0.10 | 0.08 | Heat stress transcription factor B-2c(HSF family ) |
| Os.7443.1.S1_s_at | LOC_Os03g02020 | 0.11 | 0.10 | 0.12 | 0.07 | 0.18 | 0.12 | 0.10 | 0.11 | 0.11 | 0.12 | stress responsive A/B Barrel domain containing protein, expressed |
| OsAffx.21616.1.S1_s_at | LOC_Os01g54890 | . | 0.17 | 0.11 | 0.11 | . | . | 0.17 | 0.14 | 0.11 | 0.31 | ethylene-responsive transcription factor 2, putative, expressed(AP2/EREBP family) |
| Os.12419.1.S1_at | LOC_Os03g16600 | . | 0.17 | 0.12 | 0.13 | 0.09 | 0.16 | 0.13 | 0.09 | 0.11 | 0.07 | expressed protein |
| Os.27650.1.S1_at | LOC_Os07g43560 | 0.10 | 0.17 | 0.12 | 0.08 | 0.05 | 0.13 | 0.17 | 0.17 | 0.12 | 0.08 | TKL_IRAK_DUF26-lc.24 - DUF26 kinases have homology to DUF26 containing loci, expressed |
| Os.11112.1.S1_s_at | LOC_Os08g40910 | 0.14 | 0.13 | 0.12 | 0.15 | . | 0.16 | 0.14 | 0.14 | 0.13 | 0.20 | expressed protein |
| Os.9238.2.S1_at | LOC_Os01g55270 | . | 0.20 | 0.15 | . | 0.09 | 0.19 | 0.17 | 0.12 | 0.14 | 0.09 | SGS domain containing protein, expressed |
| Os.13464.1.S1_at | LOC_Os07g44190 | . | 0.20 | 0.15 | 0.19 | 0.10 | 0.17 | 0.17 | 0.12 | 0.15 | 0.09 | h/ACA ribonucleoprotein complex subunit 4, putative, expressed |
| Os.5632.1.S1_a_at | LOC_Os05g35470 | 0.07 | 0.08 | 0.10 | 0.11 | 0.20 | 0.09 | 0.10 | 0.12 | 0.15 | 0.16 | Dienelactone hydrolase domain containing protein |
| Os.14493.1.S1_at | LOC_Os02g03670 | 0.08 | 0.08 | 0.09 | 0.08 | 0.09 | 0.14 | 0.15 | 0.14 | 0.16 | 0.09 | expressed protein |
| Os.23498.1.S1_at | LOC_Os01g69960 | . | 0.16 | 0.14 | 0.12 | 0.10 | . | 0.16 | 0.17 | 0.17 | 0.14 | expressed protein |

The DEGs identified by using the empirical criterion of more than 5-fold change and cut-off *p* < 0.05 in SAM based on three independent biological replicates. The dot in the above table means that the difference between two samples did not reach the threshold.
