# Supplementary material for: Genome-wide gene expression profiling of introgressed indica rice alleles associated with seedling cold tolerance improvement in a japonica rice background
Source: BMC Genomics. 2012 Sep 7;13:461. doi: 10.1186/z (PMC3526417; doi:10.1186/z)
Supplement: Additional file 16 — Genomic distribution of 17 introgression fragments in K354. A PowerPoint file containing information on introgressed fragments, polymorphic SSR markers used, and previously reported QTLs related to CT at the seedling stage near the introgressed regions. [file 1471-2164-13-461-S16.ppt]

## Slide 1
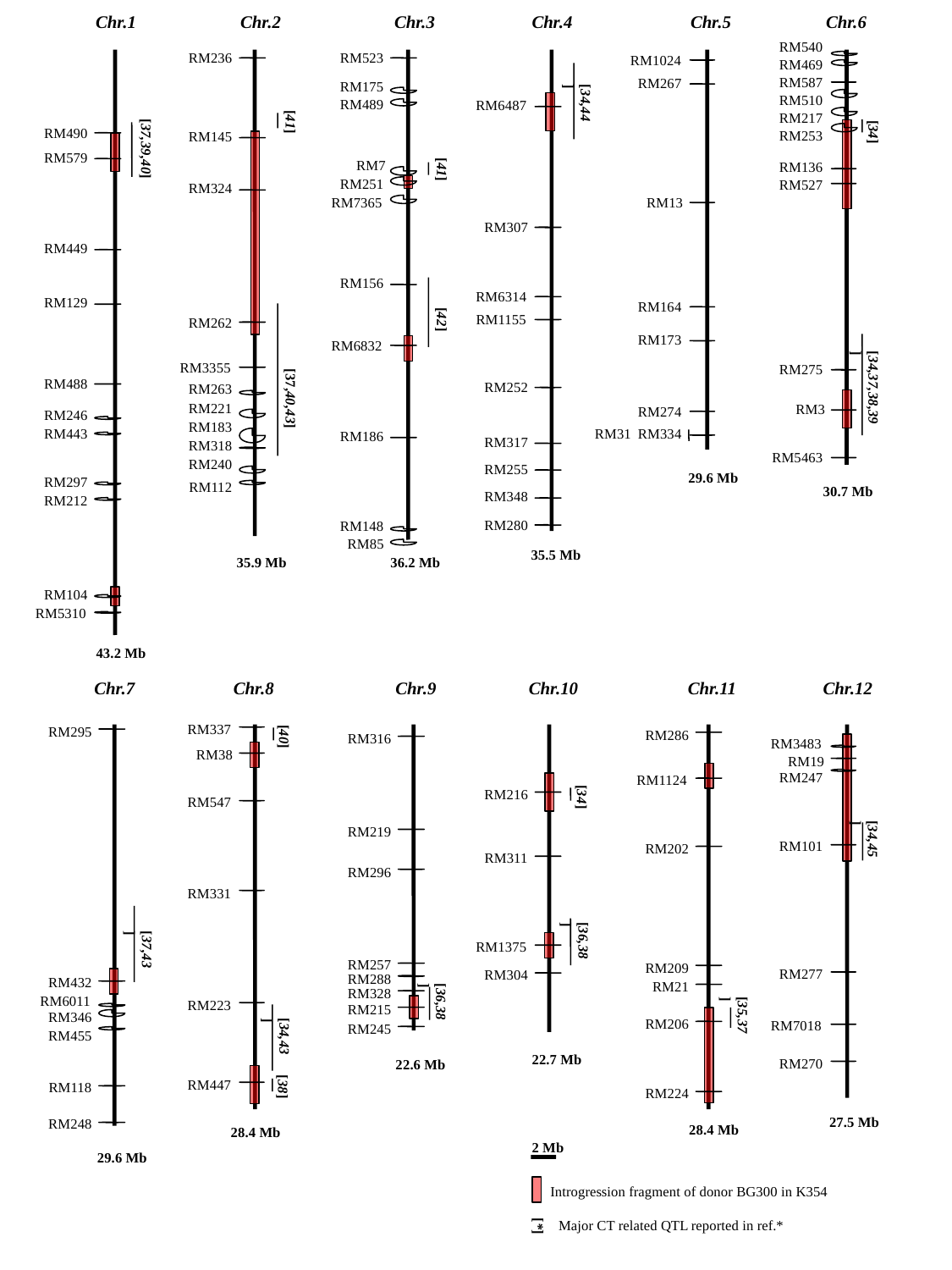

Chr.1
Chr.2
Chr.3
Chr.4
Chr.5
Chr.6
RM540
RM236
RM523
RM1024
RM469
RM587
RM267
RM175
[34,44]
RM510
RM489
RM6487
RM217
[41]
[34]
RM490
RM253
RM145
[37,39,40]
RM579
RM7
[41]
RM136
RM251
RM527
RM324
RM7365
RM13
RM307
RM449
RM156
RM6314
RM129
RM164
[42]
RM1155
RM262
RM173
RM6832
RM3355
RM275
[34,37,38,39]
RM488
RM252
RM263
[37,40,43]
RM221
RM3
RM274
RM246
RM183
RM443
RM31
RM334
RM186
RM317
RM318
RM5463
RM240
RM255
29.6 Mb
RM297
30.7 Mb
RM112
RM348
RM212
RM280
RM148
RM85
35.5 Mb
35.9 Mb
36.2 Mb
RM104
RM5310
43.2 Mb
Chr.7
Chr.8
Chr.9
Chr.10
Chr.11
Chr.12
RM337
RM295
[40]
RM286
RM316
RM3483
RM38
RM19
RM247
RM1124
[34]
RM216
RM547
[34,45]
RM219
RM101
RM202
RM311
RM296
RM331
[36,38]
[37,43]
RM1375
RM257
RM209
RM277
RM304
RM288
RM432
RM21
[36,38]
RM328
RM6011
[35,37]
RM223
RM215
RM346
[34,43]
RM206
RM7018
RM245
RM455
22.7 Mb
22.6 Mb
RM270
[38]
RM447
RM118
RM224
27.5 Mb
28.4 Mb
RM248
28.4 Mb
2 Mb
29.6 Mb
Introgression fragment of donor BG300 in K354
Major CT related QTL reported in ref.*
[*]

## Slide 2
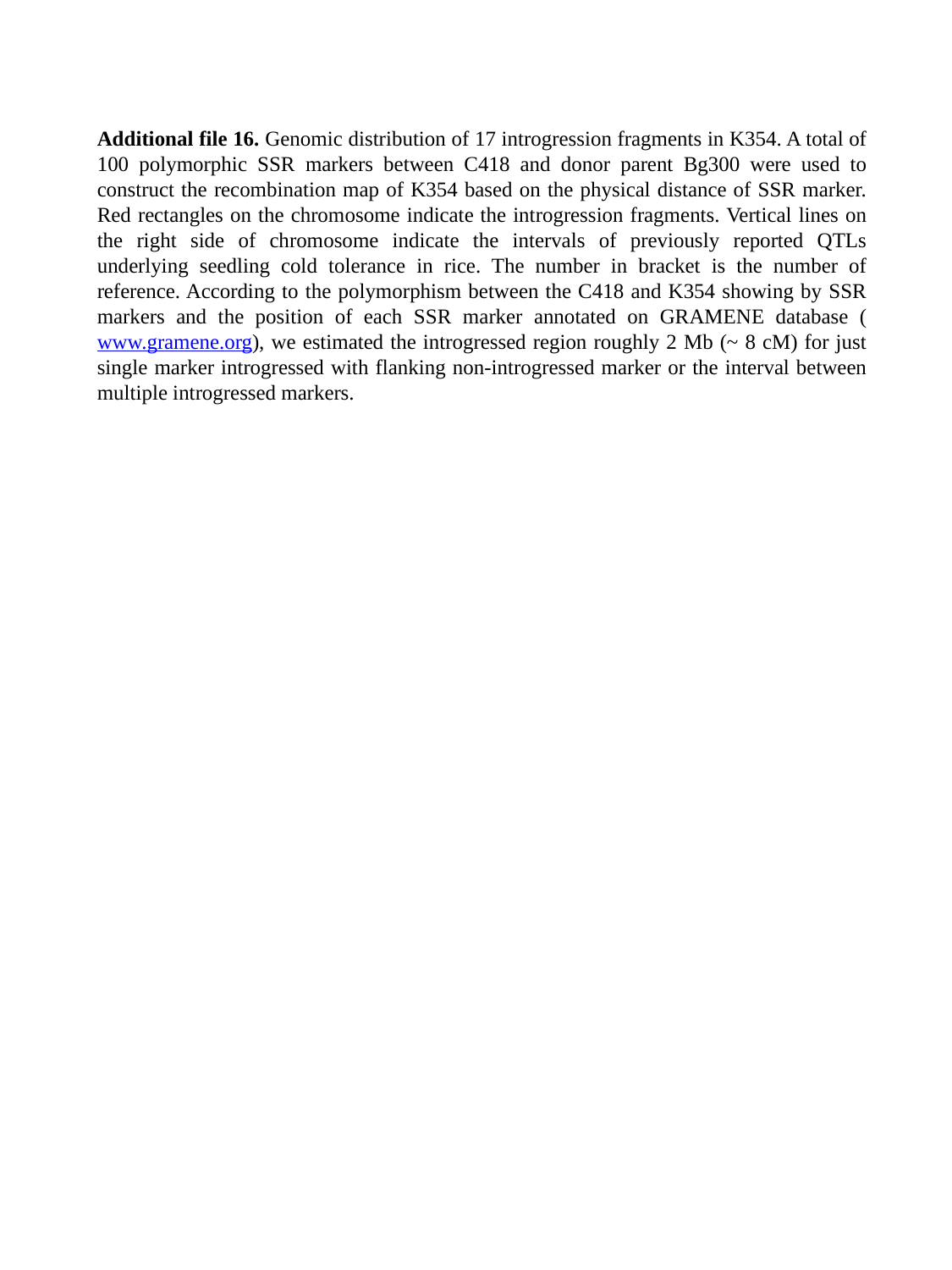

Additional file 16. Genomic distribution of 17 introgression fragments in K354. A total of 100 polymorphic SSR markers between C418 and donor parent Bg300 were used to construct the recombination map of K354 based on the physical distance of SSR marker. Red rectangles on the chromosome indicate the introgression fragments. Vertical lines on the right side of chromosome indicate the intervals of previously reported QTLs underlying seedling cold tolerance in rice. The number in bracket is the number of reference. According to the polymorphism between the C418 and K354 showing by SSR markers and the position of each SSR marker annotated on GRAMENE database (www.gramene.org), we estimated the introgressed region roughly 2 Mb (~ 8 cM) for just single marker introgressed with flanking non-introgressed marker or the interval between multiple introgressed markers.
